# Assessment and management of vitamin status in children with CKD stages 2–5, on dialysis and post-transplantation: clinical practice points from the Pediatric Renal Nutrition Taskforce

**DOI:** 10.1007/s00467-024-06303-x

**Published:** 2024-04-04

**Authors:** Caroline E. Anderson, Jetta Tuokkola, Leila Qizalbash, Matthew Harmer, Christina L. Nelms, Stella Stabouli, Barry Toole, Nonnie Polderman, An Desloovere, Jose Renken-Terhaerdt, Molly R. Wong Vega, Evelien Snauwaert, Johan Vande Walle, Dieter Haffner, Fabio Paglialonga, Rukshana Shroff, Vanessa Shaw, Larry A. Greenbaum, Bradley A. Warady

**Affiliations:** 1 University Hospital Southampton NHS Foundation Trust, Southampton, UK; 2 Human Development & Health, Faculty of Medicine, University of Southampton, Southampton, UK; 3 University of Winchester, Winchester, UK; 4 Clinical Nutrition Unit, Internal Medicine and Rehabilitation, University of Helsinki and Helsinki University Hospital, Helsinki, Finland; 5 Institute of Public Health and Clinical Nutrition, University of Eastern Finland, Kuopio, Finland; 6 Department of Medicine, Endocrinology and Clinical Nutrition, Kuopio University Hospital, Kuopio, Finland; 7 Great Northern Children’s Hospital, Newcastle Upon Tyne, UK; 8 Children’s Mercy Kansas City, Kansas City, MO USA; 9 1st Department of Pediatrics, Aristotle University, Hippokratio Hospital, Thessaloniki, Greece; 10 British Columbia Children’s Hospital, Vancouver, Canada; 11 University Hospital Ghent, Ghent, Belgium; 12 Wilhemina Children’s Hospital, University Medical Center Utrecht, Utrecht, The Netherlands; 13 Texas Children’s Hospital, Houston, TX USA; 14 Hannover Medical School, Children’s Hospital, Hannover, Germany; 15 Fondazione IRCCS Ca’ Granda Ospedale Maggiore Policlinico, Milan, Italy; 16 University College London Great Ormond Street Hospital Institute of Child Health, London, UK; 17 Emory University, Atlanta, GA USA; 18 Children’s Healthcare of Atlanta, Atlanta, GA USA

**Keywords:** Vitamins, Chronic kidney disease, Pediatric Renal Nutrition Taskforce, Clinical practice points, Children

## Abstract

Children with chronic kidney disease (CKD) are at risk for vitamin deficiency or excess. Vitamin status can be affected by diet, supplements, kidney function, medications, and dialysis. Little is known about vitamin requirements in CKD, leading to practice variation.

The Pediatric Renal Nutrition Taskforce (PRNT), an international team of pediatric kidney dietitians and pediatric nephrologists, was established to develop evidence-based clinical practice points (CPPs) to address challenges and to serve as a resource for nutritional care. Questions were formulated using PICO (Patient, Intervention, Comparator, Outcomes), and literature searches undertaken to explore clinical practice from assessment to management of vitamin status in children with CKD stages 2–5, on dialysis and post-transplantation (CKD2-5D&T). The CPPs were developed and finalized using a Delphi consensus approach. We present six CPPs for vitamin management for children with CKD2-5D&T. We address assessment, intervention, and monitoring. We recommend avoiding supplementation of vitamin A and suggest water-soluble vitamin supplementation for those on dialysis. In the absence of evidence, a consistent structured approach to vitamin management that considers assessment and monitoring from dietary, physical, and biochemical viewpoints is needed. Careful consideration of the impact of accumulation, losses, comorbidities, and medications needs to be explored for the individual child and vitamin before supplementation can be considered. When supplementing, care needs to be taken not to over-prescribe. Research recommendations are suggested.

**Supplementary Information:**

The online version contains supplementary material available at 10.1007/s00467-024-06303-x.

## Introduction

Vitamins have essential roles in bodily processes [1, 2]. Deficiencies and excesses may cause adverse clinical consequences, and chronic kidney disease (CKD) increases their risk. Vitamin status can be affected by diet, supplements, kidney function, and dialysis, among other factors.

The most recent pediatric guidelines [3, 4] for vitamin management in CKD were published over 15 years ago and are based on limited evidence. These guidelines made four key points. First, dietary intake should provide 100% of requirements for healthy children. Second, supplementation should be considered when there is inadequate intake or clinical deficiency is evident. Third, low blood concentrations may be used to confirm deficiency. Fourth, children on dialysis should receive water-soluble vitamin supplementation, if not on enteral or sip feeds (oral nutritional supplements).

Building on prior Kidney Disease Outcomes Quality Initiative recommendations [4], along with information derived from recent literature, we present a comprehensive position paper with six clinical practice points (CPPs) to address vitamin assessment, intervention, and monitoring for children with CKD stages 2–5, those on dialysis and post- kidney transplantation, all henceforth referred to as CKD. These CPPs are based on available evidence, expert opinion, and, where appropriate, extrapolation from adult studies. We address fat-soluble vitamins (A (retinol), E (tocopherol), and K (phylloquinone, menaquinone)) and water-soluble vitamins (B1 (thiamin), B2 (riboflavin), B3 (niacin), B5 (pantothenic acid), B6 (pyridoxine), B7 (biotin), B9 (folate), B12 (cobalamin), and C (ascorbic acid)). Vitamin D has been extensively addressed elsewhere and is not included in this review [5].

## Methods

The full development process, the purpose, and plans for audit and revision of the recommendations are described in a previous Pediatric Renal Nutrition Taskforce (PRNT) publication [6]. The search criteria are described below (Table S1). The PRNT working group, which included pediatric nephrologists, dietitians, and biochemists, defined the scope, formulated the questions, performed the literature review, developed the CPPs, and conducted the Delphi process. The Delphi group included nephrologists and dietitians from global children’s kidney centers.

## Development process

PICO (Patient, Intervention, Comparator, and Outcome) questions guided the development of actionable CPPs.

***A—PICO questions***

*Population/Patient:* children from birth up to 18 years of age with CKD.

*Intervention:* assessment of vitamin requirements, intake, and clinical evidence of deficiency or excess; use of supplementation and monitoring.

*Comparison:* vitamin requirements for healthy children and adults with CKD, and biochemical markers, where available *(or no comparator)*

*Outcomes:* adequate vitamin intake; avoidance of vitamin deficiencies and excesses.

***B—Proposed clinical questions for clinical practice points for children with CKD***

1. What are the vitamin requirements?
2. Which foods are the main dietary sources?
3. How do dietary modifications affect intake?
4. What are the non-dietary factors which influence vitamin status?
5. How should we approach clinical assessment and monitoring?
6. When is dietary modification or vitamin supplementation indicated?

## Literature search

Details of the literature search are described in Table S1*.* Literature up to and including a publication date of January 2023 was reviewed using PICO and related clinical questions.

## Clinical practice points

1. What are the vitamin requirements?
  1.1 The vitamin requirements for children with early CKD (stages 2–3a) should approximate those of healthy children of the same chronological age
    1.1.1 For children with advanced CKD (stage 3b–5D&T), vitamin requirements may be less or greater than those for healthy children.
    1.1.2 We suggest that the intake of vitamin A in CKD, where accumulation may occur, should not exceed the requirements of healthy children.

### Evidence and rationale

In order to understand requirements for children with CKD, we first describe the requirements for healthy children as a comparator. A summary of internationally published recommended dietary intakes of vitamins is provided in Table S2 [7–12]. Definitions of requirements can be found in Table S3.

The requirements of vitamins for children and adolescents with CKD have not been determined. Therefore, requirements for healthy children should be used as an initial guide for the assessment of adequate intake. This statement agrees with previous published guidelines. However, additional consideration has been given to advanced CKD, as discussed below, suggesting that requirements may be greater for some vitamins due to the presence of increased oxidative stress and dialysate- and medication-induced losses. Conversely, there is evidence that excess intake of some vitamins causes harm [13–15]. Children with CKD may develop clinical hypervitaminosis A due to decreased urinary excretion and absence of dialytic removal. Hence, high intake of vitamin A in CKD should be avoided [16–26]. These statements, including the restriction of vitamin A intake, are consistent with prior guidelines for children with CKD.

In children with pre-dialysis CKD, we suggest increasing intake, either via diet or supplements, if B vitamins or vitamin C intakes are lower than recommended for healthy children.

Children receiving dialysis may benefit from a higher vitamin C intake. Supplementation may be beneficial for anemia and lipid management in children on dialysis [27, 28]. However, the safe amount to ingest, to avoid the risk of systemic oxalosis, is not known [29, 30]. KDOQI [4] recommends supplementation for dialysis patients, but does not provide a specific supplementation recommendation, though does state that the safe upper limit of total intake of vitamin C is 400–1800 mg per day, depending on age. In one study, supplementation of 250 mg/day of vitamin C did not lead to hyperoxaluria [28]. The UK Scientific Advisory Committee on Nutrition and European Food Safety Authority [7, 15] have not set an upper limit for healthy children. In the absence of dietary or laboratory assessment for B vitamins, it is likely optimal to use a water-soluble multivitamin supplement in children receiving dialysis given the risk of deficiencies and low risk of excess. There are no reports of toxicity using such an approach.

2. Which foods are the main dietary source of vitamins?
  2.1 Children with CKD may obtain sufficient vitamin intake through intake of a varied diet and/or a nutritionally complete enteral formula.

### Evidence and rationale

The main dietary sources for each vitamin are shown in Table 1; some of these foods may need to be restricted to manage uremia, hyperkalemia, hyperphosphatemia, and hyperlipidemia. A varied diet can help optimize vitamin intake.

**Table 1:** Main dietary sources of vitamins and potential impact of dietary modifications

	Meat*†‡^A^	Nuts, seeds, and meat alternatives	Fish*†‡	Dairy*†‡** and egg	Fruit‡	Vegetables‡	Breads and cereals†‡	Drinks‡	Processed foods / other*†‡**
Retinol	Liver*†‡^A^		Eel*†‡	Milk*†‡, egg yolk*†, cheese*†‡**	Yellow‡	Yellow and green leafy‡		Infant and follow-on formula	Butter**, fortified vegetable spreads
E Tocopherol	Liver*†‡^A^	Almond*†‡	Sardines*†‡	Butter**, egg*† yolk,		Green and leafy vegetables‡	Wheat germs†‡, whole grains (possibly †‡)	Infant and follow-on formula	Vegetable oil
K1 Phylloquinone				Vegetable/seed fats/oils		Dark green leafy vegetables‡ (e.g., spinach, lettuce), brassica (e.g., cabbage)		Infant and follow-on formula	
K2 Menaquinones and dihydrophyl-loquinone	Liver*†‡^A^ meat*†‡, poultry*†‡, fish*†‡			Cheese*†‡**, dairy*†‡, egg yolk†**, oil, margarine shortenings			Bread (possibly †‡)	Infant and follow-on formula	Chocolate†‡, pie and pie crusts, fast foods, popcorn spices, snacks*†‡**, crackers
B1 Thiamine	Pork*†‡, small amounts in other meats	Quorn nuts*†‡, small amounts in everything	Small amounts in everything	Small amounts in everything	Small amounts in everything	Small amounts in everything	Fortified cereals and bread (possibly †‡)	Infant and follow-on formula	Small amounts in everything
B2 Riboflavin	Organ meat*†^A^ (kidney and liver), small amounts in other meats*†‡	Almond*†‡, small amounts in other nuts and seeds*†‡	Small amounts*†‡	Hard cheese*†‡**, small amounts in soft cheese, milk and other milk products*†‡ and eggs*†		Small amounts in green leafy vegetables‡, legumes†	Fortified cereals and bread (possibly †‡)	Fortified milk alternatives infant and follow-on formula	Small amounts
B3 Niacin	Liver*†‡^A^ meat*†‡	Peanuts†	Fish, especially tuna*†‡	Milk*†‡, cheese*†‡**, eggs*†			Whole grains (possibly †‡), Flour		Coffee‡, infant and follow-on formula
B5 Pantothenic acid	Meat*†‡,	Nuts†		Eggs*†, Milk*†‡	Avocado‡	Brassicas (e.g., cauliflower, cabbage, kale) and vegetables‡	Bread (possibly †‡)	Infant and follow-on formula	
B6 Pyridoxine	Meat, *†‡, poultry*†‡, liver*†‡^A^	Nuts†	Fish, crayfish*†‡			Water chestnut	Fortified cereals, wheat bran (possibly †‡)	Infant and follow-on formula	
B7 Biotin	Meat (liver^A^) *†‡, fish*†‡, poultry*†‡,			Egg yolk*†, some cheeses*†‡**		Some vegetables‡ (mushrooms), soybeans†		Infant and follow-on formula	
B9 Folate	Liver*†‡^A^	Soy†‡		Egg yolk*†‡,	Citrus and other fruits‡	Green vegetables‡, (H2018); pulses†, beetroot	Wheat germ, grains, fortified grain products (possibly †‡)	Infant and follow-on formula	
B12 Cobalamin	Animal products, meat*†‡, fish*†‡, poultry*†‡, liver*†‡^A^			Dairy products*†‡**, egg yolk*†**			Fortified food with B12, e.g., ready-to-eat breakfast cereals (possibly †‡), milk alternatives	Infant and follow-on formula	
C Ascorbic acid					Blackcurrants ‡, guava, kiwi fruit, papaya, orange, strawberries‡	Red and yellow pepper, Brussels sprouts, kale and broccoli, spinach, cauliflower, red cabbage, swede		Infant and follow-on formula, blackcurrant juice‡, orange juice‡, apple juice (fortified with vitamin C‡), fruit cordials and squashes fortified with vitamin C (values will be country specific), high fruit content cordials and squashes	

These foods may need to be restricted in case of dietary modifications for the management of *uremia, †hyperphosphatemia, ‡hyperkalemia, **hyperlipidemia; ^A^hypervitaminosis A

3. Dietary modifications and food preparation methods may impact vitamin intake.
  3.1 How do dietary modifications affect intake?

### Evidence and rationale

Dietary intake of vitamins in children with CKD has been reported to range from below to above estimated requirements for healthy children (Tables S4, S6, S10, S12, S14, S15, S16, S18, S20, S22). Diet modifications for CKD may limit vitamin-rich dietary sources (Table 1) and children with CKD are at increased risk of malnutrition, including a non-optimal vitamin status [31–33]. The risk of malnutrition may be particularly pertinent in children who have advanced CKD, including those on dialysis.

Vitamin intake can also be altered by country-specific food fortification programs and may change with cooking methods designed to reduce potassium intake. For example, boiling in water reduces vitamin content, especially of water-soluble and heat-labile vitamins [34, 35]. In addition, interactions with other nutrients may affect absorption and metabolism. The vitamin content of enteral formulas is specific at the time of manufacture, but content could change according to storage conditions [36–39].

4. What are the non-dietary factors which influence vitamin intake and status?
  4.1 Non-dietary factors, such as CKD stage, dialysis, comorbidities, and medications, should be considered when assessing and monitoring vitamin status and when planning nutritional interventions.

### Evidence and rationale

Each vitamin is considered individually below. All children, including patients with CKD, could develop vitamin deficiency due to malabsorption [40, 41]. Table 2 shows the impact of medications and how they can alter vitamin absorption, distribution, metabolism, and excretion. The most commonly prescribed medications for consideration are proton pump inhibitors (B12), furosemide (B6, C), and warfarin (B3, K, E) [42, 43].

**Table 2:** Vitamin interactions with medications [135]

Vitamin	Interactions
Vitamin A/retinol/retinoic acid	Moderate interaction: Etretinate, Tretinoin, Isotretinoin, Bexarotene – risk for retinoid toxicity Tetracycline antibiotics (e.g., doxycycline, minocycline) – increased risk of pseudotumor cerebri Anticoagulants (e.g., heparin, warfarin, dicumarol, bivalrudin, antithrombin III, argatroban, clopidogrel) – increased risk of bleeding Tocofersolan – increased absorption of vitamin A Cholestyramine – decreased absorption Rifampicin – decreased absorption Orlistat – decreased absorption
Vitamin B1/thiamin	Moderate interaction: Fluorouracil – reduced thiamine exposure
Vitamin B2/riboflavin	No moderate or major interactions No contraindications
Vitamin B3/niacin, nicotinamide	Major interaction: Simvastatin, lovastatin, rosuvastatin, cerivastatin, atorvastatin, pitavastatin—increased niacin and statin exposure with risk for myopathy or rhabdomyolysis Methotrexate – increased risk of methotrexate-related severe adverse reactions; reduced active metabolite formation; possibly reduced methotrexate efficacy Moderate interaction: Warfarin – increased risk of bleeding Insulin – decreased blood glucose lowering effect of insulin Fluvastatin, pravastatin – increased risk of myopathy or rhabdomyolysis
Vitamin B5/pantothenic acid	Moderate interaction: Azithromycin, clarithromycin, erythromycin, roxithromycin – decreases level/effect of B5 supplementation
Vitamin B6/pyridoxin, pyridoxal, pyridoxamine	Major interaction: Altretamine – reduced response to altretamine Moderate interaction: Levodopa – decreased levodopa effectiveness Furosemide – significantly increases urinary excretion
Vitamin B7/biotin	No moderate or major interactions No contraindications *Long-term use of antiseizure medications may reduce biotin levels*
Vitamin B9/folate, folic acid	Major interaction: Capecitabine – concomitant use with folic acid may result in increased fluorouracil exposure and increased risk of fluorouracil-related toxicity Pafolacianine – concurrent use may result in reduced detection of malignant lesions Methotrexate – concurrent use may result in reduced folic acid serum levels and reduced methotrexate efficacy Moderate interaction: Phenytoin – concurrent use may result in decreased folic acid serum levels; decreased phenytoin effectiveness Pyrimethamine – loss of pyrimethamine efficacy Primidone—decreased folic acid serum levels; decreased primidone effectiveness
Vitamin B12/cobalamins, cyanocobalamin methylcobalamin	Moderate interaction: Chloramphenicol – concurrent use may result in decreased efficacy of vitamin B12 when used to treat anemia Mycophenolate mofetil – increased risk of B12 deficiency due to diarrhea Proton pump inhibitors, H2 blockers, and metformin – increased risk of B12 deficiency
Vitamin C/ascorbic acid, ascorbate	Major interaction: Amygdalin – concomitant use with vitamin C may result in increased metabolism of amygdalin leading to increased cyanide levels Deferoxamine – impairment of cardiac function Erythromycin, lincomycin, streptomycin, kanamycin, doxycycline – decreased antibiotic activity Bleomycin – decreased bloemycin efficacy Moderate interaction: Indinavir (protease inhibitor/antiretroiral) – decreased indinavir plasma concentration Amphetamines (dextroamphetamine, lisdexamfetamine, benzphetamine, methamphetamine) – decreased blood levels and effectiveness of amphetamines Furosemide – significantly increases urinary excretion
Vitamin E/tocopherol, tocotrienol	Major interaction: Dicumarol – increased risk of bleeding Moderate interaction: Warfarin – increased risk of bleeding Tocofersolan – increased absorption of vitamin E Cholestyramine – decreased absorption Rifampicin – decreased absorption Orlistat – decreased absorption
Vitamin K/phylloquinone, menaquinone	Moderate interaction: Warfarin – decreased effectiveness of warfarin; reduction in INR Pau D’arco (herbal/medicinal tree bark)—reduced vitamin K effectiveness Tocofersolan – increased absorption of vitamin K Cholestyramine – decreased absorption Rifampicin – decreased absorption Orlistat – decreased absorption

Major – the interaction may be life-threatening and/or require medical intervention to minimize or prevent adverse effects

Moderate – the interaction may result in exacerbation of the patient’s condition and/or require an alteration of therapy

Vitamin A is partially excreted by the kidneys and is not removed by dialysis. The kidney tubules have a role in vitamin A catabolism, and thus vitamin A can accumulate in patients with CKD, especially those receiving dialysis [4, 16–26, 44] (Table S27). Transplant patients are considered to have CKD, and elevated levels of vitamin A may be of concern in transplant recipients, especially as kidney function declines [45, 46].

There is limited evidence that blood vitamin E concentrations are elevated in adults and children with CKD [24, 25]. There is conflicting information as to whether vitamin E is lost in hemodialysis (HD) [47–50].

Little is known about vitamin K in children with CKD. There is no evidence for an increased risk of vitamin K deficiency in CKD, though malabsorption due to gastrointestinal disease and poor intake combined with broad-spectrum antibiotics that eliminate gut bacteria producing vitamin K may cause vitamin K deficiency [51–54]. There is no evidence of vitamin K losses in dialysis; however, low serum vitamin K levels are present in patients treated with vitamin K antagonists, a therapy mainly studied in adults with CKD who exhibit side effects such as bleeding and calciphylaxis.

Many water-soluble vitamins are lost in dialysate due to their small molecular size. Only adult data is available for B complex vitamins. There is evidence for varying degrees of losses in HD for vitamins B1 (Table 3; Table S28) [55–60]. B2 [57, 60], B6 [57, 60–62], biotin [63], and folate [57, 60, 62, 64, 65]. There is evidence for varying degrees of losses in peritoneal dialysis (PD) for vitamins B2 (Table 3; Table S28) [66], B6 [57, 60, 62, 66, 67], and folate [66, 68]; losses of B1 are low [66]. There is no information on losses or levels of vitamins B3 or B5 in adults or children receiving dialysis. Losses of folate seem to be higher with high flux HD and lower with PD [64–66, 68–73]. Studies in adults have not demonstrated vitamin B12 losses in dialysis patients [57, 65, 66, 70, 71]. Table 3 summarizes the losses of B vitamins in adults receiving dialysis.

**Table 3:** Losses of water-soluble vitamins in dialysis

Vitamin	Molecular weight	Protein binding	Estimated loss in PD	Estimated loss in HD
Vitamin B1 (thiamin)	337	Albumin	Dialysate: 46 µg ± 3 mcg per session [66]	Serum reduction: 6%—[57] 20% [60] 75–82% (supplemented), 130% (unsupplemented) [56], 43.9% [59] Dialysate: 1.12 ± 0.88 mg per session [60]
Vitamin B2 (riboflavin)	376	Weak albumin	Dialysate: 832 ± 145 mcg per session [2]	Serum reduction: 7% [57], 13% [60] Dialysate: 0.28 ± 0.30 mg per session [60]
Vitamin B3 (niacin)	132	Weak binding in plasma		
Vitamin B5 (pantothenic acid)	219	Not available		
Vitamin B6 (pyridoxin)	169	Albumin Pyridoxal 5'-phosphate	Dialysate: 767 ± 111mcg per session [66]	Serum reduction: 28% ± 14% [62], 35% [57], 25.4% [60] Dialysate: 0.33 ± 0.09 mg per session [60]
Vitamin B7 (biotin)	244	Weak		Serum reduction: 30–33% [63]
Vitamin B9 (folate)	441	No	Dialysate: 107 ± 5 mcg [66]	Serum reduction: 26% ± 16% [62], 37% [57], 19% [64], 32.6% [60] Dialysate: 0.3 ± 0.18 mg [60], 37.3ug (3.6–91.5 µg) [64] per session
Vitamin B12 (cobalamin)	1355	Transcobalamin II	None reported	None reported
Vitamin C	176	No	Dialysate: 32 mg/d [77], 4.476 ± 0.543–4.659 ± 0.445 µmol/L [74]; pediatric Dialysate 4.8 ± 1.2 mg/L (unsupplemented),10.5 ± 1.8 mg/L (supplemented) [69], 50 mg/d (unsupplemented), 90 mg/d (supplemented) [79], 56 mg ± 8 mg per session [66], 40 mg per session [78], 149 µmol per 6 h per session [61]	Dialysate: 0.415 ± 0.113 µmol/L [75]; pediatric Reduction in serum 52% [76], 50% [77], 51% [81], 33% [80], 60% [82], 66.6% [60] Dialysate: 66 mg [76], 93–334 mg [77], 147.5 ± 145.5 mg [60] per session

Studies have demonstrated vitamin C losses in dialysate in children on PD [18, 74] and HD [75]. In adults, there is evidence of losses in dialysate in HD [60, 76, 77] and PD [61, 66, 69, 78, 79] as well as reduction in serum vitamin C levels in adults post-HD [60, 76, 77, 80–82] and PD [66, 68, 79, 83] (Table 3). Higher losses have been reported with higher supplementation [76]. In one study, children on HD were more likely to have low blood concentrations compared to children on PD or with CKD2–5 [84]. In adults, there are multiple studies demonstrating low blood concentrations in dialysis patients, with lower levels associated with increasing efficacy and duration of dialysis [60, 68, 69, 73, 76, 77, 79–82, 85] (Table 3). In addition, furosemide increases urinary losses of vitamin C [42].

In the transplant population, there are very limited data, with one small study suggesting no evidence of low status, and perhaps altered B vitamin metabolism with elevated B6 concentrations despite a lack of supplementation [86].

5. How should we approach clinical assessment and monitoring?
  5.1 Dietary
    5.1.1 We suggest that the vitamin intake of children with CKD should be assessed by diet history/diet records, including food and drink, formulas and nutritional supplements, and a review of medications.
    5.2.1 We suggest that the frequency of assessment of vitamin intake should be influenced by dietary modifications, child’s age, CKD stage, dialysis modality, and intake of medications that interfere with vitamin metabolism.
    5.1.3 Assessment may need to be more frequent if there are signs or symptoms of deficiency or excess.

### Evidence and rationale

Dietary assessment is clinically important given evidence of insufficient dietary intake of vitamins (Tables S4, S6, S10, S12, S14, S15, S16, S18, S20, S22) [18, 25, 28, 31, 31, 87–94]. A complete dietary assessment should include intake from food, formulas, nutritional supplements, and intake from medications [94] with consideration of the effect of food preparation on vitamin content and bioavailability. Assessment of dietary intake has been described in detail in a previous PRNT publication [94], which recommended three 24-h dietary recalls or a 3–4-day diet diary/food record. However, due to potential significant variation in daily intake [95], it is necessary to review adequacy over longer periods of time before taking action [83].

Vitamin intake should be reviewed whenever there is a major change in diet (e.g., conversion from formula to table food, adoption of a vegan diet), when a patient initiates dialysis, when there is a clinical issue that may affect vitamin balance (e.g., malabsorption, initiation of a medication that may affect vitamin levels), or when there are signs and symptoms of deficiency or excess. Dietetic assessment is best carried out by a specialized pediatric kidney dietitian or a suitably trained healthcare professional with the necessary skills and competencies. Vitamin intake based on food records or diet diaries should ideally be calculated using software with a reliable database of foods and nutrients, bearing in mind the differences in vitamin bioavailability and content due to seasonal variation.

5.2 Physical
  5.2.1 We suggest individualized evaluation for physical signs and clinical symptoms of vitamin deficiency or excess.

### Evidence and rationale

Table 4 outlines the clinical manifestations of deficiency and excess, roles of vitamins in the body, and bioavailability of vitamins. The impact of accumulation and losses are described where relevant for each vitamin below (statement 5.3). A clinical examination for signs and symptoms of vitamin deficiency or excess (Table 4) is suggested as part of routine care for children with a variety of chronic conditions [96]. In the absence of evidence, expert opinion suggests the same assessment should be undertaken for children with CKD. The frequency of assessment should be determined by clinical judgment and the outcome of relevant dietary and biochemical assessments. One challenge is that some of these signs and symptoms overlap with clinical manifestations of CKD (e.g., impaired growth, poor appetite, emesis, diarrhea, and increased fractures). Hence, the clinician must actively consider if vitamin deficiency or excess is a possible explanation for the clinical findings.

**Table 4:** Clinical manifestations of deficiency and excess, roles of vitamins in the body and their bioavailability (adapted from [136, 137])

Nutrient	Symptoms of deficiency	Symptoms of excess	Diagnostic tests (biomarkers)	Bioavailability (absorption)	Role in the body
Vitamin A	Night blindness, xerophthalmia, keratomalacia, poor bone growth, impaired resistance to infection, follicular hyperkeratosis	Hyperostosis, hepatomegaly, hepatic fibrosis, alopecia, increased cerebrospinal fluid pressure, hypercalcemia, liver damage, bone abnormalities, joint pain, alopecia, headaches, vomiting, skin desquamation	Plasma retinol	Preformed vitamin A (mainly retinol and retinyl esters) is absorbed (70–90%). The absorption of β-carotene is variable (5–65%), depending on food- and diet-related factors, genetic characteristics, and the health status of the subject	Vision; as retinal, which plays a central role in the mechanisms of photo-transduction Systemic maintenance of the growth and integrity of cells in body tissues through the action of retinoic acid, which acts as regulator of genomic expression
Thiamin (B1)	“Wernicke encephalopathy”- peripheral neuropathy, ophthalmoplegia, nystagmus, ataxia, edema, peripheral neuropathy, ataxia, various forms of dysautonomia and nystagmus, lactic acidosis. More advanced symptoms include confabulation, memory loss, psychosis; relation with sudden infant death syndrome and autism	Unknown. Urine is the main route of excretion; however, significant amounts of thiamin are excreted in feces	Thiamine pyrophosphate level, whole blood/red blood cell transketolase activation test	Thiamin absorption declines for an intake higher than 5 mg/day and absorbed thiamin is actively excreted in the urine	Precursor for coenzymes involved in carbohydrate, branched chain amino acid, and energy metabolism
Riboflavin (B2)	Sore throat, hyperaemia and edema of the pharyngeal and oral mucous membranes, cheilosis, glossitis (magenta tongue), and normochromic normocytic anemia characterized by erythroid hypoplasia and reticulocytopenia	Not sufficient clinical evidence for adverse effects of “high” riboflavin intakes; no toxic effects	24-h (preferably) or fasting urinary excretion; urinary excretion curve in relation to riboflavin intake	Absorption efficiency of 95%	Riboflavin is an integral part of the coenzymes; involved in energy metabolism, metabolic pathways, and the formation of some vitamins and coenzymes (involved in the metabolism of niacin and vitamin B6)
Niacin (B3)	Pellagra (photosensitive dermatitis, skin lesions, tongue and mouth soreness, vomiting, diarrhea, depression, and dementia). Early symptoms: weakness, loss of appetite, fatigue, digestive disturbances, abdominal pain, and irritability	Hepatotoxicity and skin flushing	Urinary excretion of niacin metabolites; less sensitive plasma niacin metabolites	The mean absorption of niacin is from about 23% to about 70%; it is lowest from cereals and highest from animal products	Precursor for nicotinamide adenine dinucleotide (NAD) and nicotinamide adenine dinucleotide phosphate (NADP)
Pantothenic acid (B5)	Mood changes, sleep, neurological, cardiac, and gastrointestinal disturbances	No known health risks with excess	Urinary pantothenic acid excretion (cut-off values are absent)	In research in six healthy young men, a mean absorption efficiency of 50% (range 40–61%) was found; further data is lacking; excreted in urine	Component of coenzyme A (CoA) and acyl-carrier proteins
Pyridoxine (B6)	Facial seborrhea, glossitis, angular stomatitis, cheilosis, mental status changes	Neuropathy, photosensitivity	Pyridoxal 5’-phosphate 4-pyridoxic acid	Absorption mainly is in the proximal small intestine through carrier-mediated, saturable transport process. Absorption efficiency of dietary riboflavin of 95%	As a part of other coenzymes involved in energy metabolism, metabolic pathways, and formation of some vitamins and coenzymes. In particular, involved in the metabolism of niacin and vitamin B6 and required by the homocysteine metabolism
Biotin (B8)	Fine scaly dermatitis, hair loss, conjunctivitis, ataxia, delayed child development	No risk for excess; there is no upper level	In adults: urinary biotin excretion; biomarkers of biotin function (urinary excretion of 3HIA) and 3HIA-carnitine; PCC activity and abundance of biotinylated MCC and PCC in lymphocytes	Free biotin is absorbed nearly completely through a saturable carrier-mediated process (passive diffusion). There is a lack of data on the level of absorption of protein-bound biotin from foods	A co-factor for the enzymes acetyl-CoA carboxylase, propionyl-CoA carboxylase, β-methylcrotonyl-CoA carboxylase and pyruvate carboxylase, which play critical roles in the synthesis of fatty acids, the catabolism of branched-chain. Fecal excretion of biotinamino acids and gluconeogenesis
Folate (B9)	Abdominal cramps, nausea, diarrhea, irritability, poor sleep, seizures, megaloblastic anemia. Neural tube defects	Masking of B12 deficiency symptoms in patients with pernicious anemia not receiving B12 Folic acid regarding sensitive cancers (incl. colorectal cancer)	Acute deficiency – low serum folic acid; chronic deficiency – low red blood cell folic acid	Natural food folate has a lower bioavailability than folic acid; the bioavailability of food folate is around 50%, i.e. half that of folic acid taken on an empty stomach; bioavailability of folic acid from fortified foods or from a supplement ingested with food is about 85%	Cofactors for enzymes involved in one-carbon metabolism. Part of the methionine cycle; producing regulators of important physiological processes
Cobalamin (B12)	Megaloblastic anemia neurological problems, cardiovascular symptoms, methylmalonic aciduria	No adverse effects with excess intake	Blood concentrations of cobalamin, holotranscobalamin, and the metabolites methylmalonic acid and total homocysteine	Absorption of cobalamin is variable, depending on the dietary source, the amount of cobalamin ingested, the ability to release cobalamin from food and the proper functioning of the intrinsic factor system; absorption of 40% (a conservative estimate). The highest losses occur through the feces (in the bile). If the circulating cobalamin exceeds the cobalamin binding capacity of the blood, the excess is excreted in the urine	Coenzyme for metabolic reactions
Vitamin C (ascorbic acid)	“Scurvy”- osmotic diarrhea, gingival bleeding, perifollicular hemorrhage, long bone changes (arthropathy) Decreased immunity, impaired wound healing, localized pain, and CVD	Massive doses predispose to kidney stones; nausea, abdominal pain; rebound scurvy when massive doses stopped	White blood cell ascorbate concentration, radiographic evidence of long bone widening	About 80% for an intake of about 100 mg/day	Enzyme cofactor for biochemical reactions catalyzed oxygenases. A role in the biosynthesis of collagen. Essential for the synthesis of carnitine and catecholamines. Involved in the metabolism of cholesterol to bile acids antioxidant
Vitamin E	Hemolytic anemia in premature infants; fat malabsorption causes deficiency; hyporeflexia and spinocerebellar and retinal degeneration	Bleeding, impaired leukocyte function	Plasma alpha-tocopherol	In presence of fat 75%	Important antioxidant protecting lipid cell walls from oxidative damage and resulting dysfunction, particularly in erythrocytes and nerve cells. Role in platelet aggregation Enhances cell-mediated and humoral immunity
Vitamin K	Bleeding tendency	No adverse effects on high prophylactic doses	Deficiency can be demonstrated by a vitamin K-responsive increase in prothrombin time or partial thromboplastin time	Absorption and transport in the body is complex; absorption is dependent on the kind of meal; fat has a positive effect on the absorption; it is not possible to estimate precisely an average absorption from the diet	Cofactor of c-glutamyl carboxylase that catalyzes the carboxylation of glutamic acid residues into c-carboxyglutamic acid residues in vitamin K-dependent proteins, which convert them into their active forms. These Gla-proteins are involved in different physiological processes, including blood coagulation or bone mineralization

5.3 Biochemical
  5.3.1 Routine biochemical assessment of vitamin status is not indicated.
  5.3.2 We suggest biochemical assessment when there are signs or symptoms of deficiency or excess, and in children with:
    unexplained hypercalcemia or elevated intracranial pressure—assess vitamin A.
    unexplained macrocytic anemia—assess vitamins folate and B12.
  5.3.3 Consider biochemical assessment if there are risk factors for deficiencies or excess in children:
    receiving peritoneal dialysis—assess vitamins C, B2, B6, and folate.
    receiving hemodialysis—assess vitamins C, B1, B5, B6, biotin, and folate.
    taking medication that may interfere with vitamin metabolism and/or absorption - assess applicable vitamins
  5.3.4 Biochemical assessment should ideally be undertaken when fasted, when not acutely ill, and pre-hemodialysis session.
    measure C-reactive protein (CRP) when assessing vitamin C and B6 levels.

### Evidence and rationale

Routine biochemical assessment of vitamin status is not indicated unless there is evidence of malabsorption. Previous guidelines recommended routine biochemical assessment, but there is no evidence in the existing literature that routine monitoring is necessary. This statement differs from previously published guidelines based on current evidence. Vitamin biochemical assessment, as described in Table 4, is, however, appropriate in patients with signs and symptoms compatible with vitamin deficiency or excess. The frequency of monitoring vitamin biochemical status should be determined using clinical judgment. Individual vitamin considerations are discussed below.

### Vitamin A

Hypercalcemia secondary to hypervitaminosis A has been reported in pediatric and adult dialysis patients [23, 44, 97–100]. Retinol is a form of vitamin A; elevated retinol levels increase osteoclastic breakdown of bone and decrease osteoblastic bone formation [26]. Vitamin A toxicity may also cause increased intracranial pressure [101]. Hence, biochemical assessment for vitamin A excess, along with evaluation of vitamin intake, should be considered in a patient with advanced CKD and unexplained hypercalcemia or raised intracranial pressure.

### Vitamin E

There is no evidence that deficiency or excess from elevated vitamin E levels occurs in CKD. Thus, we do not recommend vitamin E levels be measured.

### Vitamin K

Vitamin K-dependent proteins play an important role in coagulation and deficiency can lead to increased bleeding [9, 11, 12]. Hence, assessment for vitamin K deficiency by measuring prothrombin time (PT) (Table 4) is appropriate when there is unexplained bleeding or bruising, especially in a patient with risk factors for deficiency (statement 4.0).

### Vitamin B complex

Routine measurement of B complex vitamins is not indicated in children with CKD; suboptimal as well as good biochemical status has been described in the literature [18, 24, 25, 86–89, 92, 102–112], as shown in Tables S5, S7, S11, S13, S17, S19, S21, and S23. The reference normal values for the B complex vitamins are often based on limited data in children and thus must be interpreted with caution.

Measurement of B12 and folate levels in patients with CKD is most recommended as part of the evaluation of unexplained macrocytic anemia. For patients with a borderline B12 level, an elevated methylmalonic acid (MMA) level supports a diagnosis of B12 deficiency; MMA is usually elevated in CKD patients, but high or disproportionally elevated levels of MMA for CKD stage may suggest B12 deficiency [113–115]. Treatment of folic acid deficiency may mask the macrocytic anemia and worsen the neurological manifestations of B12 deficiency, and thus B12 levels should be monitored in patients treated for folic acid deficiency [83].

Noteworthy is recognition that acute inflammation, assessed by the measurement of CRP, lowers the level of vitamin B6 [116]. Hence, CRP should be determined when measuring B6 and the level of B6 should be repeated if it is initially low in the setting of an elevated CRP level.

### Vitamin C

Routine measurement of vitamin C is not indicated in children with CKD [25, 28, 74, 75, 84, 89]. Plasma vitamin C can be measured if there is clinical suspicion of deficiency or excess (Table 4). Since oxalate is a byproduct of vitamin C metabolism (statement 6.2), plasma or urine oxalate can also be measured if vitamin C excess is suspected [29, 117]. Plasma vitamin C should be measured in a fasting state, and before dialysis in HD patients. Moreover, acute inflammation lowers the level of vitamin C [116]. Thus, CRP should be measured when measuring vitamin C. If the CRP is elevated, the vitamin C concentration should be repeated once the CRP level has normalized.

6. When is dietary modification or supplementation indicated?
  6.1 Intervention is indicated:
    if dietary assessment suggests a risk of deficiency or excess.
    if clinical and/or biochemical evidence suggests a risk of deficiency or excess.
  6.2 Fat-soluble vitamins
    6.2.1 Dietary sources rich in vitamin A should be limited in children with CKD.
    6.2.2 If nutritional supplements and formulas are indicated, consider one with a lower vitamin A content.
    6.2.3 If a multivitamin supplement is indicated, consider one with a low or zero vitamin A content.
    6.2.4 Avoid routine vitamin E and K supplementation unless a comorbidity predisposing to deficiency is present.
  6.3 Water-soluble vitamins
    6.3.1 In the case of low water-soluble vitamin intake, consider dietary modification and/or vitamin supplementation.
    6.3.2 Water-soluble vitamin supplementation (particularly vitamins C, B6, and folate) may be needed in children on dialysis because of dialysate losses.
    6.3.3 Water-soluble vitamin supplementation may be needed in children while taking medications that interfere with vitamin metabolism.
    6.3.4 We suggest folate and vitamin B12 supplementation may be required in children with macrocytic anemia and biochemical evidence of deficiency.
    6.3.5 Consider the benefits versus risks of vitamin C supplementation due to its metabolism to oxalate.

### Evidence and rationale

There are limited studies describing the dietary intake and serum concentrations of vitamins in children with CKD, and results vary with CKD stage. Reported intakes range from lower to higher than the estimated requirements for healthy children, with higher intakes in children receiving vitamin supplements [18, 18, 23, 25, 28, 31, 31, 87–89, 89–93]. Similarly, serum concentrations range from below normal to above normal [16, 18, 18, 20–26, 74, 75, 84, 87, 89, 92, 102, 104, 105, 107, 109, 111, 118, 119]. Risk of excess intake for those vitamins that accumulate with declining kidney function is greatest when patients receive vitamin supplements (Tables S4, S6, S10, S12, S14, S15, S16, S18, S20, S22). These practice points are consistent with previous guidelines but include additional guidance on when and how to adjust dietary intake and prescribe vitamin supplements.

### Fat-soluble vitamins

Supplementation of fat-soluble vitamins is not recommended.

### Vitamin A

There is a high risk of vitamin A toxicity, due to decreased excretion. A diet with high amounts of vitamin A-rich foods can far exceed the recommended intake for healthy children. Hence, the nutritional evaluation in children with CKD should include assessment for high intake of vitamin A from dietary sources (e.g., consumption of liver). Formulas, nutritional supplements, and vitamin supplements may also lead to excessive intake of vitamin A [18, 22, 23, 89]. Consequently, it has been proposed that children with CKD be prescribed no or the lowest vitamin A content possible when using nutritional or vitamin supplements, with the goal to limit intake to the recommended intake for healthy children, or lower [3].

### Vitamin E

There are no studies of vitamin E supplementation in children with CKD. Supplementation studies in adult patients are limited with inconclusive results. Supplementation is not recommended, and excess intake should be avoided [83]. A meta-analysis of supplementation trials suggested a dose-dependent relationship of increased all-cause mortality (0.4%) with high-dose vitamin E supplementation [120].

### Vitamin K

There is no evidence to support the routine supplementation of vitamin K in patients with CKD [121]. It is important to avoid vitamin K supplementation in patients receiving warfarin for anticoagulation [83]. Children receiving frequent antibiotic therapy should be carefully monitored for risk of deficiency.

### Water-soluble vitamins

Water-soluble vitamins are commonly lost in dialysate. Sufficient dietary intake may not occur due to overall poor intake, selective eating, and dietary modifications due to CKD. Eating a varied diet rich in water-soluble vitamins, with due regard for necessary dietary modifications, is encouraged. Given the cost and limits of biochemical assessment, use of a balanced water-soluble multivitamin supplement is a practical option in children with poor dietary intake for sustained periods of time, increased losses due to dialysis, or chronic furosemide therapy (Table 2). Since commercial vitamin supplements may provide a far higher intake than requirements, it is recommended that children with CKD are monitored for clinical evidence of vitamin excess. Children receiving enteral or oral formulas will likely not need a multivitamin supplement.

### Vitamin B complex

There are no trials looking at the effects of vitamin B complex intake on serum levels in children with CKD. Study results in adults vary, with patients receiving dialysis having low or adequate blood concentrations with and without supplementation (Table S28) [56, 61, 66, 68, 69, 73, 79, 122–125]. Hyperhomocysteinemia, which may be secondary to folate or B12 deficiency, is common in children with CKD, including post-transplant [107, 108]. Although there are reports of improved hematologic parameters [126], homocysteine levels [127], and oxidative stress indices [128] with folate supplementation in children with CKD, there is no evidence for empirical supplementation with pharmacological doses of folate in the absence of biochemical-proven deficiency.

When folate supplementation is initiated, consider vitamin B12 supplementation if biochemical measurements of B12 are not available [83].

### Vitamin C

There are no body stores of vitamin C and thus blood concentrations decrease quickly after stopping supplementation [66]. Supplementation may increase levels, although the evidence of clinical benefit is limited [83]. Supplementation of children may not be sufficient to normalize blood vitamin C concentration in those who receive dialysis [25, 28, 75]. In adults, there are multiple studies demonstrating improvement in vitamin C blood concentrations with supplementation [68, 69, 77, 79–82].

In one pediatric study, intravenous supplementation with 250 mg vitamin C post-HD decreased total and LDL cholesterol [27]. In adult kidney transplant recipients, lower vitamin C concentrations were associated with a higher risk of graft failure [129].

Functional iron deficiency is an important cause of anemia in CKD patients, and vitamin C may increase iron bioavailability [130]. Studies have demonstrated a benefit of oral vitamin C supplementation on anemia indices in children with CKD 4–5D [28] and adults on PD [78]; anemia indices have also improved with intravenous vitamin C supplementation in adult HD patients [131, 132]. Excess supplementation and intake of vitamin C may, however, lead to elevated blood oxalate concentrations. Due to decreased oxalate excretion in the urine, plasma oxalate levels increase in children and adults receiving dialysis, but the effect on tissue levels of oxalate is uncertain. There are case reports of systemic oxalosis in children and adults [29, 30]. Most studies did not see an elevation in serum oxalate levels with a maximum vitamin C supplementation of 250 mg per day [28, 131, 133, 134], while some did with supplementation of 500 mg per day [82]. Thus, supplementation should be provided with caution and total intake of vitamin C from diet and formulas should not exceed 250 mg per day [28, 30].

## Results of the Delphi survey

Thirty-one responses were received via an electronic Delphi survey, comprising 18 dietitians and 13 pediatric nephrologists across 16 countries. Delphi respondents are listed under Acknowledgements as “Participants in Delphi survey.”

Of the six clinical practice recommendation statements, overall, a 91.1% consensus was achieved with a “strongly agree” or “agree” response, 6.6% had a “neutral” response, 1.7% “disagree,” and 0.5% “strongly disagree” response. All but one statement met the stipulated 70% or higher level of consensus. The one statement received a response rate of 65% agreement (statement 5.3.1). The respondents queried the need for vitamin D biochemical assessment and suggested routine biochemical assessment in chronic disease. Vitamin D has been extensively reviewed in previous publications and was not in scope for this review. The most recent vitamin D review reference is noted in the paper. Evidence covered in the rationale does not currently support routine biochemical vitamin assessment and a research recommendation has been made to explore criteria for biochemical assessment. The taskforce team carefully reviewed all of the statements in light of these responses; none required significant change.

## Summary of statements

A summary of statements is provided in Table 5.

**Table 5:** Summary of clinical practice point statements

	Statements
*1.0 What are the vitamin requirements?*	1.1 The vitamin requirements for children with early CKD (stages 2–3a) should approximate those of healthy children of the same chronological age 1.1.1 For children with advanced CKD (stage 3b–5D&T), vitamin requirements may be less or greater than those for healthy children 1.1.2 We suggest that the intake of vitamin A in CKD, where accumulation may occur, should not exceed the requirements of healthy children
*2.0 What are the main dietary sources?*	2.1 Children with CKD may obtain sufficient vitamin intake through intake of a varied diet and/or a nutritionally complete enteral formula
*3.0 How do dietary modifications affect intake?*	3.0 Dietary modifications and food preparation methods may impact vitamin intake
*4.0 What are the non-dietary factors which influence vitamin intake and status?*	4.1 Non-dietary factors, such as CKD stage, dialysis, comorbidities, and medications should be considered when assessing and monitoring vitamin status and when planning nutritional interventions
*5.0 How should we approach clinical assessment and monitoring?*	***5.1—Dietary*** 5.1.1 We suggest that the vitamin intake of children with CKD should be assessed by diet history/diet records, including food and drink, formulas and nutritional supplements, and a review of medications 5.2.1 We suggest that the frequency of assessment of vitamin intake should be influenced by dietary modifications, child’s age, CKD stage, dialysis modality, and intake of medications that interfere with vitamin metabolism 5.1.3 Assessment may need to be more frequent if there are signs or symptoms of deficiency or excess
	***5.2—Physical*** 5.2.1 We suggest individualized evaluation for physical signs and clinical symptoms of vitamin deficiency or excess
	***5.3—Biochemical*** 5.3.1 Routine biochemical assessment of vitamin status is not indicated 5.3.2 We suggest biochemical assessment when there are signs or symptoms of deficiency or excess, and in children with: - unexplained hypercalcemia or elevated intracranial pressure—assess vitamin A - unexplained macrocytic anemia—assess folic acid and vitamin and B12 5.3.3 Consider biochemical assessment if there are risk factors for deficiencies or excess in children: - receiving peritoneal dialysis—assess vitamins C, B2, B6, and folic acid - receiving hemodialysis—assess vitamins C, B1, B5, B6, biotin, and folic acid - taking medication that may interfere with vitamin metabolism and/or absorption—assess applicable vitamins 5.3.4 Biochemical assessment should ideally be undertaken when fasted, when not acutely ill, and pre-hemodialysis session - measure C-reactive protein (CRP) when assessing vitamin C and B6 levels
*6.0 When is dietary modification or supplementation indicated?*	6.1 Intervention is indicated: - if dietary assessment suggests a risk of deficiency or excess - if clinical and/or biochemical evidence suggests a risk of deficiency or excess ***6.2 Fat-soluble vitamins*** 6.2.1 Dietary sources rich in vitamin A should be limited in children with CKD 6.2.2 If nutritional supplements and formulas are indicated, consider one with a lower vitamin A content 6.2.3 If a multivitamin supplement is indicated, consider one with a low or zero vitamin A content 6.2.4 Avoid routine vitamin E and K supplementation unless a comorbidity predisposing to deficiency is present ***6.3 Water-soluble vitamins*** 6.3.1 In the case of low water-soluble vitamin intake consider dietary modification and/or vitamin supplementation 6.3.2 Water-soluble vitamin supplementation (particularly vitamins C, B6, and folic acid) may be needed in children on dialysis as a result of dialysate losses 6.3.3 Water-soluble vitamin supplementation may be needed in children while taking medications that interfere with vitamin metabolism 6.3.4 We suggest folic acid and vitamin B12 supplementation may be required in children with macrocytic anemia and biochemical evidence of deficiency 6.3.5 Consider the benefits versus risks of vitamin C supplementation due to its metabolism to oxalate

## Research recommendations

There is a need for well-designed longitudinal observational studies of the dietary intake of vitamins and the serum status of vitamins over time, accompanied by randomized controlled studies designed to help determine the vitamin requirements for children with CKD. The most pressing topics to explore are:

### Intake/requirements

- Determination of the requirements for optimal health, specifically:

- vitamin K—the potential for improved vascular and bone health.
- vitamin A—benefits and risks of limiting intake below reference requirements.
- vitamin C—safe upper level regarding oxalate accumulation, kidney stone formation, and nausea
- vitamin C and B complex—losses on dialysis, effects of diuretics, and urine losses.
- impact of transplantation on vitamin status.
- development of dietary intake assessment methods that reflect true intake and/or dietary risk.
- exploration of the relationship between outcome measures such as improved cognition, growth, and quality of life, with improved vitamin status.

### Biochemical status

- Assessment methods for vitamin biochemical status.
- Determination of the safe serum concentrations of:

- vitamin A
- vitamin C with respect to oxalate

- Assessment of status in children not receiving vitamin supplements.

### Supplementation

- Development of optimal age-based multivitamin supplement.
- Determination of the amount of supplementation needed to achieve normal serum concentrations, body stores, and functions.
- Exploration of the benefit and long-term safety of:

- vitamin E supplementation and other antioxidant vitamins in improving clinical outcomes such as cardiovascular risk, disease progression, and mortality.
- vitamin K supplementation with long-term use of antibiotics and for bone health.
- vitamin C supplementation on oxalosis, cardiovascular risk and/or erythrocyte lifespan, lipid levels, and anemia management.
- vitamin B3 supplementation on lipid levels and phosphate management.
- folate supplementation on anemia management.

## Conclusions

Vitamins have essential roles in body processes. CKD can alter vitamin intake and biochemical status and thus requirements may differ in children with CKD compared with their healthy peers. We present clinical practice points designed to address clinical issues pertaining to vitamin status in children with CKD as a key aspect of their nutritional management. Ensuring sufficient vitamin intake and status is essential not only to prevent deficiencies and excess, but also to help ensure long-term health. Consuming rich dietary sources of most vitamins should be promoted. Dietary modifications necessary for urea, phosphate, potassium, and lipid management may reduce vitamin intake, but should not compromise vitamin status.

### Supplementary Information

Below is the link to the electronic supplementary material.

Supplementary file1 (DOCX 123 KB)
