# Supplementary material for: Assessment and management of vitamin status in children with CKD stages 2–5, on dialysis and post-transplantation: clinical practice points from the Pediatric Renal Nutrition Taskforce
Source: Pediatr Nephrol. 2024 Apr 4;39(10):3103–24. doi: 10.1007/s00467-024-06303-x (PMC11349803; doi:10.1007/s00467-024-06303-x)
Supplement: Supplementary file 1 — Supplementary file1 (DOCX 123 KB) [file 467_2024_6303_MOESM1_ESM.docx]

**Table S1** Search term strategy used in the literature review

| **1** | Kidney Disease | Kidney Failure | Renal Failure | Renal Insufficiency | Kidney Injury | Kidney dysfunction | Chronic Kidney Disease (CKD) | Chronic Kidney Failure (CKF) |
| --- | --- | --- | --- | --- | --- | --- | --- | --- |
|  | Chronic Renal Failure (CRF) | End Stage Renal Disease | End Stage Renal Failure | Renal Replacement Therapy | Dialysis | Pre Dialysis | Peritoneal Dialysis | Hemodialysis |
|  | Haemodialysis | CAPD continuous ambulatory peritoneal dialysis | APD automated peritoneal dialysis | Uraemia | Uremia | Transplant |  |  |

| **2** | Diet | Dietary | Nutrition | Food | Feed | Intake | Requirements | Nutritional support |
| --- | --- | --- | --- | --- | --- | --- | --- | --- |
|  | Dietary management | Dietary advice | Dietary restriction | Supplementation | Dietitian | Dietician | Enteral nutrition | Oral intake |
|  | Diet recall | Mixed diet | Formula | Supplement(s) | Assessment | Serum | Plasma | Red blood cell |

| **3** | Serum | Plasma | Red blood cell | Food record | Food frequency questionnaire (FFQ) | 24-h recall (24 hour) | Diet history | Deficiency |
| --- | --- | --- | --- | --- | --- | --- | --- | --- |
|  | Toxicity | Adequacy | Erythrocyte | Urinary excretion | Urinary  Status | Nutriture | Depletion | Repletion |
|  | Live (storage) | Tissue | Prothrombin time |  |  |  |  |  |

| **4** | Vitamin A  , | Retinol | retinoic acid | Retinol equivalents | a-carotene | b-carotene | Retinol binding protein | Vitamin E |
| --- | --- | --- | --- | --- | --- | --- | --- | --- |
|  | Tocopherol | Tocotrienol | Vitamin K | Phylloquinone | Menaquinone | Vitamin C | Ascorbic acid | Ascorbate |
|  | Vitamin B1 | Thiamine | Thiamin | Transketolase activation | Vitamin B2 | Riboflavin | EGRAC (erythrocyte glutathione reductase activation coefficient) | Vitamin B3 |
|  | Niacin | Nicotinamide | Niacin equivalents | NAD(P) | N-methyl | Nicotinamide | NMH | MPCX (methyl pyridone carboxamide) |
|  | Typtophan | Vitamin B5 | Pantothenic acid | Pantothenate | Vitamin B6 | Pyridoxin | Pyridoxine | Pyridoxal |
|  | Vitamin B7 | Biotin | Vitamin B9 | Folate | Folic acid | Vitamin B12 | Cobalamins | Cyanocobalamin |
|  | Methylcobalamin |  |  |  |  |  |  |  |

**Search methods**

1980 – January 2023 English language

Medline / PubMed, Embase, Cochrane library, Cinahl, manual searching

Given the paucity of studies in this field, all publications, including meta-analyses, randomized controlled trials, prospective studies, retrospective studies, case studies (irrespective of patient numbers) have been considered.

Adult studies were used when pediatric studies were lacking.

**Table S2.** A summary of internationally published recommended dietary intakes of vitamins

| ***Vitamin*** | ***A*** | ***E*** | ***K*** | ***C*** | ***B1*** | ***B2*** | ***B3*** | B5 | ***B6*** | B7 | ***B9*** | B12 |
| --- | --- | --- | --- | --- | --- | --- | --- | --- | --- | --- | --- | --- |

| **Organisation/Body** | **EAR/RNI/RI/PRI/DRI** | **EAR/RNI/RI/PRI/DRI** | **EAR/RNI/RI/PRI/DRI** | **EAR/RNI/RI/PRI/DRI** | **EAR/RNI/RI/PRI/DRI** | **EAR/RNI/RI/PRI/DRI** | **EAR/RNI/RI/PRI/DRI** | **EAR/RNI/RI/PRI/DRI** | **EAR/RNI/RI/PRI/DRI** | **EAR/RNI/RI/PRI/DRI** | **EAR/RNI/RI/PRI/DRI** | **EAR/RNI/RI/PRI/DRI** |
| --- | --- | --- | --- | --- | --- | --- | --- | --- | --- | --- | --- | --- |
| **Recommendations:** |  |  |  |  |  |  |  |  |  |  |  |  |
| **Age (months):** |  |  |  |  |  |  |  |  |  |  |  |  |
| **0 thru 4 months** | 180-501 | 2.2-5 | 2-5 | 15-40 | 0.2-0.3 | 0.3-0.4 | 2-6.6 | 1.7-2 | 0.1 | 4-10 | 40-60 | 0.25-0.5 |
| **4 thru 6 months** | 190-400 | 2.8-4 | 2-5 | 15-40 | 0.2-0.3 | 0.3-0.4 | 2-6.6 | 1.7-3 | 0.1-0.3 | 4-10 | 40-80 | 0.25-1.4 |
| **7 thru 11 months** | 200-400 | 2.5-6 | 2.5-10 | 15-50 | 0.1-0.3 | 0.3-0.4 | 1.6-6.6 | 1.7-3 | 0.3 | 6-10 | 40-80 | 0.35-1.5 |
| **1 year** | 200-400 | 3.6-5 |  | 15-35 | 0.1-0.5 | 0.4-0.7 | 1.6-6.6 | 2-4 | 0.3-0.6 | 8-20 | 50-150 | 0.4-1.5 |
| **2 years** | 200-400 |  | 12.1/11.5-30 | 15-35 | 0.1-0.5 | 0.4-0.7 | 1.6-6.6 | 2-4 | 0.4-0.6 | 8-20 | 50-150 | 0.4-1.5 |
| **3 years** | 200-400 | 3.2-6 | 12.1/11.5-30 | 15-35 | 0.1-0.5 | 0.4-0.8 | 1.6-6.6 | 2-4 | 0.4-0.6 | 8-20 | 50-150 | 0.4-1.5 |
| **4 years** | 200-400 | 3.2-6 | 12.1/11.5-30 | 20-35 | 0.1-0.7 | 0.4-0.8 | 1.6-6.6 | 3-4 | 0.4-0.6 | 10-25 | 75-200 | 0.7-2 |
| **5 years** | 200-400 | 3.2-9 | 18.6/18.4-55 | 20-35 | 0.1-0.7 | 0.5-0.8 | 1.6-6.6 | 3-4 | 0.5-0.7 | 10-25 | 75-200 | 0.7-2 |
| **6 years** | 200-400 | 4.9-9 | 18.6/18.4-55 | 20-40 | 0.1-0.7 | 0.5-0.8 | 1.6-6.6 | 3-4 | 0.5-0.7 | 10-25 | 75-200 | 0.7-2 |
|  | 250-500 | 4.9-9 | 18.6/18.4-55 | 20-40 |  |  |  | 3-4 | 0.5-1 | 10-25 |  | 0.8-2.5 |
| **7 years** | 250-500 | 4.9-9 | 27.4/27.5-55 | 20-40 | 0.1-0.9 | 0.8-1.1 | 1.6-16 | 3-4 |  | 10-25 | 110-300 | 0.8-2.5 |
| **8 years** |  | 5.6/5-9 | 27.4/27.5-55 | 20-40 | 0.1-0.9 | 0.8-1.1 | 1.6-16 |  | 0.5-1 | 10-25 | 110-300 | 0.8-2.5 |
| **9 years** | 250-600 | 5.6/5-9 | 27.4/27.5-60 | 20-50 | 0.1-0.9 | 0.8-1.1 | 1.6-16 | 3-5 | 0.5-1 | 10-35 | 110-600 | 0.8-2.5 |
| **10 years** | 330-600 | 5.6/5-9 | 27.4/27.5-60 |  | 0.1-1.2/1.1 | 0.8-1.4/1.2 | 1.6-16 | 3-5 | 0.8-1 | 10-35 | 110-600 | 1-.35 |
| **11 years** | 330-600 | 5.6/5-13/11 | 41.1-43.2-60 | 22/35-70 | 0.1-1.2/1.1 | 0.8/1.0-1.4/1.2 | 1.6-16 | 3-5 | 0.8-1.3/1.2 | 10-35 | 150-600 | 1-3.5 |
| **12 years** | 330-600 | 6.4/5.3-13/11 | 41.1-43.2-60 | 22/35-70 | 0.1-1.2/1.1 | 0.8/1.0-1.4/1.2 | 1.6-16 | 3-5 | 0.8-1.3/1.2* | 10-35 | 150-600 | 1-4 |
| **13 years** | 330-600 | 6.4/5.3-13/11 | 41.1-43.2-60 | 22.35-70 | 0.1-1.2/1.1 | 0.8/1.0-1.4/1.2 | 1.6-16 | 3-6 | 0.8-1.3/1.2* |  | 150-600 | 1-4 |
| **14 years** | 330-7/900 | 6.4/5.3-13/11 | 41.1-43.2-75 | 22/35-75 | 0.1-1.4/1.1 | 0.8/1-.7/1.2 | 1.6-18/15 | 3-6 | 0.8-1.3/1.2* | 10-35 | 150-800 | 1-4.5 |
| **15 years** | 330-7/900 | 6.4/5.3-13/11 | 61.5/55.6-75 | 25-90/100 | 0.1-1.4/1.1 | 0.8/1-.7/1.2 | 1.6-18/15 | 3-6 | 1.1/0.8-1.5-1.2* | 10-40 | 150-800 | 1-4.5 |
| **16 years** | 330-7/900 | 8/6.1-13/11 | 61.5/55.6-75 | 25-90/100 | 0.1-1.4/1.1 | 0.8/1-.7/1.2 | 1.6-18/15 | 3-6 | 1.1/0.8-1.5-1.6* | 10-40 | 150-800 | 1-4.5 |
| **17 years** | 330-7/900 | 8/6.1-13/11 | 61.5/55.6-75 | 25-90/100 | 0.1-1.4/1.1 | 0.8/1-.7/1.2 | 1.6-18/15 | 3-6 | 1.1/0.8-1.5-1.6* | 10-40 | 150-800 |  |
| **18 years** |  | 8/6.1-13/11 |  |  |  |  |  |  | 1.1/0.8-1.5-1.6* |  |  |  |

*Not SACN (Vitamins: A, k, B7, B9 and B12 = mcg; Vitamins: E, C, B1, B2, B3, B5, B6 = mg)

**Sources: ^^[[1]](#footnote-1)^^**

**Table S3**. Definitions of requirements.

| **EAR** | Estimated Average requirement | Average daily intake level to meet 50% of needs |
| --- | --- | --- |
| **RNI** | Recommended nutrient intake | EAR + 2 standard deviations (SD) to meet the need of most healthy individuals in age and sex-specific populations (97.5%) |
| **AH** | Apparently healthy | Absence of disease based on clinical signs and symptoms of micronutrient deficiency or excess and normal function on laboratory values and physical examination |
| **PNI** | Protective nutrient intake | Amount greater than RNI which may be protective against a specific health or nutrition risk of public health relevance |
| **UL** | Upper tolerable nutrient intake level | Maximum intake from food, water and supplements that is unlikely to pose a risk of adverse health effects from excess in most individuals. |
| **NE** | Nutrient excess | Above the highest level at which no observed adverse effects of biological significance are found |
| **NOAEL** | No observed adverse effect level |  |
| **LOAEL** | Lowest observed adverse effect level |  |
| **OI** | Optimal intake | Establish the function of interest and the desired level of function. |

**Sources of terminology: ^^[[2]](#footnote-2)^^**

**Table S4.** Summary of studies with vitamin A dietary intake in children with CKD

| **Author, year, & location** | **Type of study** | **Patients** | **Age** | **N** | **Intervention** | **Outcome measures** | **Findings** | **Potential bias / limitations** |
| --- | --- | --- | --- | --- | --- | --- | --- | --- |
| Coleman and Watson 1992  UK | Prospective | CCPD | 0.3-12.6 yrs | 7 | Renal Vitamin supplement given | Diet levels  3 day food diary | Adequate intake of vitamin A from supplements and diet. 93% Rx diet | No control group |
| Foreman JW et al 1996 USA | Prospective | eGFR 20-75 | 1.5-11.9 yrs | 120 | 401 4 day Food Records (baseline, 2,4 and 6 months) | Dietary Intake | Mean vitA intake normal except in 11+ -yr-olds |  |
| Kriley & Warady 1991 USA | Prospective | PD vs. Controls | 6.1-12.9 years | 8 | Evaluation of serum levels in patients compared to controls | Serum levels  Weighted 3 day food diaries | Intakes slightly above DRI's. 130% Rx | Children on supplements but no vitamin A in them |
| Manickavasagar et al, 2015 UK | Cohort | 25 CKD 2-3, 35 in CKD 4-5, 23 dialysis, 22 post-transplant |  | 105 | Evaluation of serum levels and diet | Dietary vitamin A intake was assessed using a food diary. | 53 % had vitamin A intake above the Reference Nutrient Intake (RNI) value. Children receiving supplemental feeds compared with diet alone had higher vitamin A intake (p = 0.02) and higher serum ROH (p < 0.001).  6% 2 x Rx, 38% on diet, 62% diet+sup. | Length of food diary. Single centre. |
| My Thuc LT 2019 Vietnam | Cross-sectional | PD | 8.5 =/- 4.2 yrs | 31 | Evaluation of nutritional deficiencies | Dietary Levels  24-h dietary questionnaire | Intakes of vitamin A below mean, 2 patients with clinical deficiency supplemented, vitamin A levels not assessed.  Dietary intake 63% of DRI | Not based on food records.  35.5% malnourished. No vitA plasma levels, but diagnosed 2 cases with clinical vitamin A deficiency due to signs of ulcerative lesions in eyes?!? |
| Tuokkola J et al 2021 | Retrospective / cross-sectional | Chronic dialysis | 0-16 yrs | 33 | Evaluation of serum vitamin A and other V/M levels in comparison to dietary intakes | Serum levels and nutritional intakes | Dietary intake of vitamin A moderate 73% Rx idet. 166% on feeds.. 11% overall, | Only child with normal serum vitamin A levels had pancreatic insufficiency and fat malabsorption |
| Warady BA et al 1994 USA | Prospective | PD vs control | Infants 7 to 16 months | 7 | serum vitamin levels and dietary (formula and supplement) intakes | Serum levels | Elevated vitamin A intakes.  Diet alone 134% requirements, |  |

**Table S5.** Summary of studies with vitamin A biochemical status in children with CKD

| **Author, year, & location** | **Type of study** | **Patients** | **Age** | **N** | **Intervention** | **Outcome measures** | **Findings** | **Potential bias / limitations / notes** |
| --- | --- | --- | --- | --- | --- | --- | --- | --- |
| Becker-Cohen R 2012 |  | Boys with Dent's disease |  | 8 | Evaluation of vitamin A status | Serum Vitamin A levels | Children with kidney impairment had normal RBP levels and children with symptoms had low levels |  |
| Casey CE et a; 1981, USA | Prospective, controlled study | 9 HD 2 PD | 6-19 yrs | 9 | Serum samples | Elevated vs non elevated vitamin A levels | Elevated levels compared to control group, statistically significant |  |
| Fassinger N et al 2010 USA | Retrospective chart review | ESRD  HD, PD | < 12 or > 12 yr age groups | 75 | ROH, RBP, TTR values evaluated | Elevated levels | Elevated levels of all 3 | Factors such as feeds, supplementation not controlled for |
| Harris et al, 2021 USA | Retropsective | CKD 4-5 |  | 58 | Vitamin A formula vs none | Serum levels | Formula fed children with higher calcium and vitamin A levels |  |
| Joyce et al, 2020  UK | Retrospective cross-sectinal | CKD, non-D | median age 8.97 yrs | 112 | Evaluation of serum levels | Serum levels | 81% with elevated vitamin A levels | Not well defined ages, someon supplements,some not, Hard to quantify |
| Joyce, et al. 2018 UK | Restrospective | PD | Median age 11.4 yrs | 47 | Evaluation of serum levels | Serum levels | 94% elevated vitamin A levels in dialysis patients | Not consistent supplementation |
| Kriley & Warady 1991 USA | Prospective | PD vs. Controls |  | 8 | Evaluation of serum levels in patients compared to controls | Serum levels  Weighted 3 day food diaries | Vitamin A levels significantly higher in dialysis patients compared to controls, intakes slightly above DRI's. | Children on supplements but no vitamin A in them |
| Lipkin&Lenssen 2008 USA | Retrospective, descriptive | AKI on RRT “o to stem cell transplantatin | <19yrs, median 4.7 yrs | 19 | Evaluation of serum vitamin A levels via molar vitamin A and RBP levels | Serum levels | 17/19 with elevated levels, 7 with possible toxicity symptoms. 89% | Lack of initial vitamin A levels, no control for past medical hx.  intakes from enteral and parenteral nutrition able to be calculated, typically above goal intake, all received a vitamin, 18/19 with vitamin A in it unless on PN, levels more than 3 times normal (vit A) in children <20 kg |
| Lughetti 2008 Italy | Prospective, controlled | CRF + controls | 11 =/- 4.6 yrs | 9 / 8 | Evaluation of lipid levels in CRF children | Lipid levels  Antioxidant levels of lipoproteins | Elevated lipid levels in children with CKD compared to controls; also lower antioxidants levels in lipoproteins including B-carotene | Small number.  specific to B carotene in lipoproteins, not serum vitamin A levels |
| Manickavasagar et al, 2015 UK | Cohort | 25 CKD 2-3, 35 in CKD 4-5, 23 dialysis, 22 post-transplant |  | 105 | Evaluation of serum levels and diet | Serum retinoid measures included retinol (ROH), its active retinoic acid (RA) metabolites [all-trans RA (at-RA) and 13-cis RA] and carrier proteins [retinol-binding protein-4 (RBP4) and transthyretin (TTR)]. | Higher serum ROH (p < 0.001).,Increased ROH seen in CKD stage 2. For every 10 ml/min/1.73 m(2) fall in estimated glomerular filtration rate (eGFR), there was a 13 % increase in ROH. RBP4 levels were increased in CKD 3-5 and dialysis patients. The lowest ratios of ROH:RBP4 were seen in dialysis compared with CKD 2-3 (p = 0.03), suggesting a relative increase in circulating RBP4. Serum ROH, RBP4 and at-RA were associated with serum calcium. On multivariable analysis RBP4 levels and alfacalcidol dose were significant predictors of serum calcium (model R (2) 32 %) in dialysis patients. | Length of food diary. Single centre.  hypercalcemia common in hypervitaminosis A |
| Sethi SK 2009 India | Retropsective | Dents disease |  | 3 | Evaluation of responsiveness to vitamin A supplementation | Improvement in night blindness | All 3 responsive to high dose vitamin A supplementation | Review of patients, no controls |
| Tuokkola J et al 2021 | Retrospective | Chronic dialysis | 0-16 yrs | 33 | Evaluation of serum vitamin A and other V/M levels in comparison to dietary intakes | Serum levels and nutritional intakes | Dietary intake of vitamin A moderate, Elevated levels in 97% of patients | Only child with normal serum vitamin A levels had pancreatic insufficiency and fat malabsorption |
| Warady BA et al 1994 USA | Prospective | PD vs control | Infants 7 to 16 months | 7 | Serum vitamin levels and dietary (formula and supplement) intakes | Serum levels | Elevated vitamin A levels despite no supplementation | Small number  patients on water soluble supplements without vitamin A, normal range 0.7-2.8, mean level 4.6 |
| Zwolinska et al, 2006 Poland | prospective controlled | Pre-D mod (32),  More sever e CRF (14),  HD  controls |  | 46 in CRFgps  21 HD | Plasma, erythrocyte and dialysate levels | Plasma, erythrocyte levels compared to controls and each group | Plasma A levels low, lowest in HD pts, erythrocyte A levels same as controls | Oxidative stress may relate to low antioxidant vitamin levels |
| Zwolinska et al, 2009 Poland | Cohort | PD  Contorls8 | 13.2 yrs =/- 3.5 yrs | 10  27 | Plasma, dialysate and erythrocyte levels | Plasma levels of antioxidants compared to controls | Decreased plasma levels of A compared to controls, loss of antioxidants in dialysate | Oxidative stress |

**Table S6.** Summary of studies with vitamin E dietary intake in children with CKD.

| **Author, year, & location** | **Type of study** | **Patients** | **Age** | **N** | **Intervention** | **Outcome measures** | **Findings** | **Potential bias / limitations / notes** |
| --- | --- | --- | --- | --- | --- | --- | --- | --- |
| Coleman and Watson 1992 UK | Cohort | CKD5D-PPD | 0.3-12.6 | 7 | Plasma and 3 day recall | Dietary intake 128% Rx diet and 190% + diet  Plasma | Adequate intake | Small |
| Tuokkola 2012 | Cross-sectional | PD, HD | Median 1.2 (IQR 0.8-8.3) yrs | 33 |  | Dietary intake 3-day food records | Dietary intake 190% (156-207)% on feeds, 128 (102-211)% food alone, 169 (102-229)% food + supplements | Small number of children who were not on feeds |

**Table S7.** Summary of studies with vitamin E biochemical status in children with CKD

| **Author, year, & location** | **Type of study** | **Patients** | **Age** | **N** | **Intervention** | **Outcome measures** | **Findings** | **Potential bias / limitations / notes** |
| --- | --- | --- | --- | --- | --- | --- | --- | --- |
| Drukker 1988 Israel | Cohort | PreD 10  HD 10  PD 10  PostT 10 | Mean 10.9 (3-19yrs) | 50 | Normal plasma levels | Plasma concentration | **All groups similar, and** normal **range** | Corrected for lipid concentrations, and used fating samples - that theoretically may influence serum concentratoins. |
| Lughetti 2008 Italy | Cohort | PreD 9  control 8 | Mean 11 yrs | 17 |  | Plasma | **No difference** | Small |
| Nemeth et al 2000 Hungary | Trial | HD 10 | Mean 15.2 yrs (7017yrs) | 10 | Vit E supplementation | Markers of oxidative stress | The addition of vitamin E ameliorated the increase in oxidative stress seen with erythropoietin treatment, and resulted in a larger incremental change of haemoglobin and haematocrit | Small. Status not reported. |
| Naseri 2015 Iran | Cohort | PD 12 HD 25 | Mean 13.8 yrs (1.6-25Yrs) | 43 |  | Serum concentrations, and mortality. | Serum vitamin E more likely low than normal or high with **7**2% low.  21% were within the normal reference range, and 7% were hgh .  HD pts (n=25), 84% had low concentrations, 12% normal, 4% high.  PD pts (67% low, 33% normal).  High mortality rate. Those with low vitamin E status did not have a higher incidence of death (p=0.175). | As defined as <3, 6, 5ug/ml for children, teenagers, and adults, respectively)  (>9, 10, 18 ug/mL, respectively |
| Tahzib et al 1999 USA | Trial | FSGS 11 Non FSGS 9  11 PreD  9 controls | Mean 12.9 yrs |  | 200 IU vitamin E BD for 3 months. | Proteinuria | improvement in proteinuria in FSGS group, no change in other aetiology group. | Small. Status not reported. |
| Tuokkola 2021 Finland | Cross-sectional  Restrospective | *PD, HD* | Median 1.2 (IQR 0.8-8.3) yrs | 33 | Supplementation | Serum alpha-tocopherol | On feeds: 39.0 (26.5-45.5) µmol/L; eating food: 31.0 (26.0-45.0) µmol/L | Small number of children who were not on feeds |
| Zwolinska et al, 2009 Poland | Cohort | PD 10  Control 27 | Mean 13.2 yrs | 37 | Low plasma PD, nomral HD | Plasma conc | Loss of vitamin E in the ultrafiltrate. | Vitamin A concentrations are low - questions validity of study as not typical of CKD. |
| Zwolinska 2006 Poland | Cohort | HD 21  PD 27 | Mean 12.9 yrs | 48 | Low plasma and RBC in HD, normal CKD compared to controls. | Plasma conc | Pre-D pts reported lower plasma and RBC concentrations cf. controls. | Vitamin A concentrations are low - questions validity of study as not typical of CKD. |

**Table S8.** Summary of studies with vitamin E interventions in children with CKD

| **Author, year, & location** | **Type of study** | **Patients** | **Age** | **N** | **Intervention** | **Outcome measures** | **Findings** | **Potential bias / limitations / notes** |
| --- | --- | --- | --- | --- | --- | --- | --- | --- |
| Nemeth et al 2000 Hungary | Trial | HD 10 | Mean 15.2 yrs | 10 | Vit E supplementation | Markers of oxidative stress | The addition of vitamin E ameliorated the increase in oxidative stress seen with erythropoietin treatment, and resulted in a larger incremental change of haemoglobin and haematocrit | Small. Status not reported. |
| Tahzib et al 1999 USA | Trial | FSGS 11 Non FSGS 9  11 PreD  9 controls | Mean 12.9 yrs |  | 200 IU vitamin E BD for 3 months. | Proteinuria | improvement in proteinuria in FSGS group, no change in other aetiology group. | Small. Status not reported. |

**Table S9.** Summary of studies with vitamin K interventions in children with CKD

| **Author, year, & location** | **Type of study** | **Patients** | **Age** | **N** | **Intervention** | **Outcome measures** | **Findings** | **Potential bias / limitations / notes** |
| --- | --- | --- | --- | --- | --- | --- | --- | --- |
| Paglialonga, 2016, Italy | Cohort | CKD  active NS / prev CVC thrombosis  Controls prev CVc thrombosis | M 10.6yrs (1.2-15.3Yrs); 11.8 (6.1-17.3) | 9 and 8 (38 enrolled) | Vit K antagonists, warffarin | Overall CVC survival | CVC survival sig higher in warffarin group (100vs 60% and 83.3 va 16.7% p=<0.05, who were treated with Vit K | Pilot study, small numbers,, unknown incidence of Central vein thrombosis; standard group are the control group; sublavian line use; |

**Table S10.** Summary of studies with vitamin B1 dietary intake in children with CKD

| **Author & year, Titles and journal, type of study** | **Location** | **Patients** | **Age** | **N** | **Intervention** | **Outcome measures** | **Findings** | **Potential bias / limitations** | **Notes** |
| --- | --- | --- | --- | --- | --- | --- | --- | --- | --- |
| Don T 2010  Cross-sectional | New Zeeland | PD | 7.8 +-5.3 yrs | 12 | 1 patient with 2.2 mg supplementation/day | Dietary intake 3-day food records, | Total median intake 245% of DRI (range 128-408%), diet alone 82% | Small study. |  |
| Kim H et al 2014  Cross-sectional | South Koreal | CKD 2-5 | 10-19 yrs | 19, 10 GFR > 75 mL/min , 9 < 75 mL/min |  | Dietary intake, 3-day food records | Intake % of RDA in GFR ≥ 75 90+-10%, in GFR < 75 60+-20% |  | Dietary recommendations intakes for Korea . Ad libitum diet, ht z scores -0.2 +/- 0.8 , wt z score -0.2 +/- 1.3 |
| Kriley & Warady 1991  Cross-sectional | USA | APD, CAPD | 5.3 yrs | 8 patients + 6 healthy controls | Daily supplementation 1.5 mg/day | Dietary intake, 3-day weighed food records | Dietary intake median 62% of RDA, with suppl 598% | Small study. Diet not described (use of sip feeds?) |  |
| My Thuc LT 2019  Cross-sectional | Vietnam | PD | 8.5 +- 4.2 yrs | 31 |  | Dietary intake, 24-h dietary questionnaire | Intake 120% of DRI | 35.5% of patients malnourished |  |
| Pereira AM 2000  Prospective | Brazil | 4 IPD, 19 CAPD, 7 HD | 9.3 +- 7.4 yrs | 30 | Supplementation | Dietary and supplemental intake adequacy, 3-day food records;  anthropometry | Intakes % of RDA diet PD 69 +- 29, HD 58+-35, with suppl PD 616+-191, HD 558+-57 | No blood levels |  |
| Warady B 1994  Cross-sectional | USA | PD | 11.4+-2.6 mo | 7 | Supplementation 0.6 mg/day | Dietary intake 3-day food records | Dietary intake 135% of RDA, diet + suppl 277% | Small study |  |
| Tuokkola 2021  Cross-sectional | Finland | 22 P D, 11 HD | Median 1.2 (IQR 0.8-8.3) yrs | 33 | Supplementation | Dietary intake 3-day food records | on feeds 135% of RNI, feeds + suppl 160% food 81%, food + suppl 363% | Small number of children who were not on feeds |  |

**Table S11.** Summary of studies with vitamin B1 biochemical status in children with CKD

| **Author & year, Titles and journal, type of study** | **Location** | **Patients** | **Age** | **N** | **Intervention** | **Outcome measures** | **Findings** | **Potential bias / limitations** | **Notes** |
| --- | --- | --- | --- | --- | --- | --- | --- | --- | --- |
| Don T 2010  Cross-sectional | New Zeeland | PD | 7.8 +-5.3 yrs | 12 | 1 patient with 2.2 mg supplementation | Red-cell concentration | Mean levels above reference range | Small study. |  |
| Kriley & Warady 1991  Cross-sectional | USA | APD, CAPD | 5.3 yrs | 8 patients + 6 healthy controls | Daily supplementation 1.5 mg | Blood concentration | Greater stores in patients than in controls | Small study. Diet not described (use of sip feeds?) |  |
| Warady B 1994  Cross-sectional | USA | PD | 11.4+-2.6 mo | 7 | Supplementation 0.6 mg/day | Activation of erythrocyte transketolase | Mean levels below normal, 14.2% +- 5.1 (normal 20%), indicating greater stores. | Small study |  |
| Tuokkola J 2021 | Finland | 22 PD, 11 HD | Median 1.2 (IQR 0.8-8.3) yrs | 33 | Supplementation | fasted red blood-cell concentration | Mean levels above reference range for both on feeds and eating food. Median (IQR) On feeds 120.0 (77.0-164.0), on food 122.0 (67.0-170.5) nmol/L | Small number of children who were not on feeds |  |

**Table S12.** Summary of studies with vitamin B2 dietary intake in children with CKD

| **Author & year, Titles and journal, type of study** | **Location** | **Patients** | **Age** | **N** | **Intervention** | **Outcome measures** | **Findings** | **Potential bias / limitations** | **Notes** |
| --- | --- | --- | --- | --- | --- | --- | --- | --- | --- |
| Don T 2010  Cross-sectional | New Zeeland | PD | 7.8 +-5.3 yrs | 12 | 1 patient with 4 mg supplementation | Dietary intake 3-day food records | Intake 272% (range 147-513%) of RDA | Small study. |  |
| Kim H et al 2014  Cross-sectional | South Koreal | CKD 2-5 | 10-19 yrs | 19, 10 GFR > 75 mL/min , 9 < 75 mL/min |  | Dietary intake, 3-day food records | Intake % of RDA in GFR ≥ 75 80+-20%, in GFR < 75 50+-10% |  | Dietary recommenedations intakes for Korea . Ad libitum diet, ht z scores -0.2 +/- 0.8 , wt z score -0.2 +/- 1.3 |
| Kriley & Warady 1991  Cross-sectional | USA | APD, CAPD | 5.3 yrs | 8 patients + 6 healthy controls | Daily supplementation 1.7 mg/day | Dietary intake, 3-day weighed food records | 128% controls, 82% diet, 232% diet + suppl | Small study. Diet not described (use of sip feeds?) |  |
| My Thuc LT 2019  Cross-sectional | Vietnam | PD | 8.5 +- 4.2 yrs | 31 |  | Dietary intake, 24-h dietary questionnaire | Intake 100% of DRI | 35.5% of patients malnourished |  |
| Pereira AM 2000  Prospective | Brazil | 4 IPD, 19 CAPD, 7 HD | 9.3 +- 7.4 yrs | 30 | Supplementation | Dietary and supplemental intake adequacy, 3-day food records;  anthropometry | Intakes % of RDA diet PD 79+-54, HD 62+-24, with suppl PD 259+-103, HD 228+-23 | No blood levels |  |
| Warady B 1994  Cross-sectional | USA | PD | 11.4+-2.6 mo | 7 | Supplementation 0.6 mg/day | Dietary intake 3-day food records | Diet 157% of RDA, diet + suppl 271% | Small study |  |
| Tuokkola 2021  Cross-sectional | Finland | PD, HD | Median 1.2 (IQR 0.8-8.3) yrs | 33 | Supplementation | Dietary intake 3-day food records | on feeds 182% of RNI, feeds + suppl 200%  food 66%, food + suppl 192% | Small number of children who were not on feeds |  |

**Table S13.** Summary of studies with vitamin B2 biochemical status in children with CKD

| **Author & year, Titles and journal, type of study** | **Location** | **Patients** | **Age** | **N** | **Intervention** | **Outcome measures** | **Findings** | **Potential bias / limitations** | **Notes** |
| --- | --- | --- | --- | --- | --- | --- | --- | --- | --- |
| Don T 2010  Cross-sectional | New Zeeland | PD | 7.8 +-5.3 yrs | 12 | 1 patient with 2.2 mg supplementation | Red-cell concentration | Mean levels above reference range | Small study. | No correlation between dietary  intake and the biochemical status |
| Kriley & Warady 1991  Cross-sectional | USA | APD, CAPD | 5.3 yrs | 8 patients + 6 healthy controls | Daily supplementation 1.5 mg | Blood concentration | Greater stores in patients than in controls | Small study. Diet not described (use of sip feeds?) |  |
| Warady B 1994  Cross-sectional | USA | PD | 11.4+-2.6 mo | 7 | Supplementation 0.6 mg/day | Activation of erythrocyte transketolase | mean levels within normal range, 0.88% +- 0.19 (normal range 0.9+-0.08) | Small study |  |

**Table S14.** Summary of studies with vitamin B3 dietary intake in children with CKD

| **Author & year, Titles and journal, type of study** | **Location** | **Patients** | **Age** | **N** | **Intervention** | **Outcome measures** | **Findings** | **Potential bias / limitations** | **Notes** |
| --- | --- | --- | --- | --- | --- | --- | --- | --- | --- |
| Kim H et al 2014  Cross-sectional | South Koreal | CKD 2-5 | 10-19 yrs | 19, 10 GFR > 75 mL/min , 9 < 75 mL/min |  | Dietary intake, 3-day food records | Intake % of RDA in GFR ≥ 75 90+-10%, in GFR < 75 70+-20% |  | Dietary recommenedations intakes for Korea . Ad libitum diet, ht z scores -0.2 +/- 0.8 , wt z score -0.2 +/- 1.3 |
| My Thuc LT 2019  Cross-sectional | Vietnam | PD | 8.5 +- 4.2 yrs | 31 |  | Dietary intake, 24-h dietary questionnaire | Intake 143.3% of DRI | 35.5% of patients malnourished |  |
| Pereira AM 2000  Prospective | Brazil | 4 IPD, 19 CAPD, 7 HD | 9.3 +- 7.4 yrs | 30 | Supplementation | Dietary and supplemental intake adequacy, 3-day food records;  anthropometry | Intakes % of RDA diet PD 71+-27, HD 63+-33, with suppl PD 236+-66, HD 205+-28 | No blood levels |  |
| Tuokkola 2021  Cross-sectional | Finland | 22 P D, 11 HD | Median 1.2 (IQR 0.8-8.3) yrs | 33 | Supplementation | Dietary intake 3-day food records | on feeds 123% of RNI, feeds + suppl 159% food 118%, food + suppl 247% | Small number of children who were not on feeds |  |

**Table S15.** Summary of studies with vitamin B5 dietary intake in children with CKD

| **Author & year, Titles and journal, type of study** | **Location** | **Patients** | **Age** | **N** | **Intervention** | **Outcome measures** | **Findings** | **Potential bias / limitations** | **Notes** |
| --- | --- | --- | --- | --- | --- | --- | --- | --- | --- |
| Pereira AM 2000  Prospective | Brazil | 4 IPD, 19 CAPD, 7 HD | 9.3 +- 7.4 yrs | 30 | Supplementation | Dietary and supplemental intake adequacy, 3-day food records;  anthropometry | Intakes % of RDA diet PD 92+-17, HD 72+-14, with suppl PD 185+-30, HD 150+-31 | No blood levels |  |

**Table S16.** Summary of studies with vitamin B6 dietary intake in children with CKD

| **Author & year, Titles and journal, type of study** | **Location** | **Patients** | **Age** | **N** | **Intervention** | **Outcome measures** | **Findings** | **Potential bias / limitations** | **Notes** |
| --- | --- | --- | --- | --- | --- | --- | --- | --- | --- |
|  |  |  |  |  |  |  |  |  |  |
| Don T 2010  Cross-sectional | New Zeeland | PD | 7.8 +-5.3 yrs | 12 | 1 patient with 4 mg supplementation | Dietary intake 3-day food records | total median 410% of DRI (range 63-1288%), diet alone 55% | Small study. |  |
| Foreman JW 1996, prospective | USA | GFR 20-75 | range 1.5-11.9 yrs, mean 6.1+- 3.2 yrs | 120 children, 401 food records |  | 4-day Food records at enrollment and at 2, 4, and 6 months | Mean B6 intake was low in all except 1-3-yr-olds |  | Well controlled food records with scales and measuring cups provided for families. |
| Kim H et al 2014  Cross-sectional | South Koreal | CKD 2-5 | 10-19 yrs | 19, 10 GFR > 75 mL/min , 9 < 75 mL/min |  | Dietary intake, 3-day food records | Intake % of RDA in GFR ≥ 75 90+-10%, in GFR < 75 70+-20% |  | Dietary recommenedations intakes for Korea . Ad libitum diet, ht z scores -0.2 +/- 0.8 , wt z score -0.2 +/- 1.3 |
| Kriley & Warady 1991  Cross-sectional | USA | APD, CAPD | 5.3 yrs | 8 patients + 6 healthy controls | Daily supplementation 10 mg/day | Dietary intake, 3-day weighed food records | Intake % of RDA 107% controls, 97% diet, 893% diet + suppl* | Small study. Diet not described (use of sip feeds?) |  |
| Pereira AM 2000  Prospective | Brazil | 4 IPD, 19 CAPD, 7 HD | 9.3 +- 7.4 yrs | 30 | Supplementation | Dietary and supplemental intake adequacy, 3-day food records;  anthropometry | Intakes % of RDA diet PD 72+-22, HD 56+-26, with suppl PD 249+-54, HD 203+-36 | No blood levels |  |
| Stockberger R 1987  Prospective, intervention | USA | CAPD |  | 8 | Supplementation 1-10 mg/day for 1 yr | Dietary intake, 3-day food records | Dietary intake without supplementation below RDA. | Small study | Losses in dialysate 3.7+-2.5 % of dietary intake; in urine 0.9+-0.8 % of diet. No PLP detected in dialysate. |
| Warady B 1994  Cross-sectional | USA | PD | 11.4+-2.6 mo | 7 | Supplementation 0.5 mg/day | Dietary intake 3-day food records | Intake from diet 60% of RDA, diet + suppl 139% | Small study |  |
| Tuokkola 2021  Cross-sectional | Finland | 22 PD, 11 HD | Median 1.2 (IQR 0.8-8.3) yrs | 33 | Supplementation | Dietary intake 3-day food records | Intake on feeds 164% of RNI, feeds + suppl 222% food 94%, food + suppl 690% | Small number of children who were not on feeds |  |

**Table S17.** Summary of studies with vitamin B6 biochemical status in children with CKD

| **Author & year, Titles and journal, type of study** | **Location** | **Patients** | **Age** | **N** | **Intervention** | **Outcome measures** | **Findings** | **Potential bias / limitations** | **Notes** |
| --- | --- | --- | --- | --- | --- | --- | --- | --- | --- |
| Don T 2010  Cross-sectional | New Zeeland | PD | 7.8 +-5.3 yrs | 12 | 1 patient with 4 mg supplementation | whole blood concentration of pyridoxine-5-  phosphate | mean levels above reference range | Small study. | No correlation between dietary  intake and the biochemical status |
| Joyce T 2018a  retrospective | UK | 15 HD, 13 PD | 9.4 (2.4, 14.3) yrs | 28 | 15 children on enteral feeds / oral nutritional supplements. Eight with supplementation 0.5-14.5 mg/day | whole blood pyridoxal 5′-  phosphate | 61% of children had blood B6 concentrations above reference range. Median (IQR). 372.9 (246.5, 446.8) nmol/L. |  |  |
| Joyce T 2018b  retrospective | UK | 28 HD, 19 PD | median 11.4 (2.8, 14.4) yrs | 47 (6 with B6) |  | 12/47 received oral nutritional supplement or enteral feed | All vitamin B6 levels above reference range. | Only 6 measurements of vit B6! Dietary intake not reported |  |
| Kriley & Warady 1991  Cross-sectional | USA | APD, CAPD | 5.3 yrs | 8 patients + 6 healthy controls | Daily supplementation 10 mg/day | Plasma pyridoxal phosphate | 84 in controls, 167 in patients | Small study. Diet not described (use of sip feeds?) |  |
| Stockberger R 1987  Prospective, intervention | USA | CAPD |  | 8 | Supplementation 1-10 mg/day for 1 yr | Blood total B6 | Plasma PLP levels lower than those reported in healthy children (reference values??)., plasma B6 concentrations higher than in healthy children. | Small study | Losses in dialysate 3.7+-2.5 % of dietary intake; in urine 0.9+-0.8 % of diet. No PLP detected in dialysate. |
| Warady B 1994  Cross-sectional | USA | PD | 11.4+-2.6 mo | 7 | Supplementation 0.5 mg/day | pyridoxal phosphate | Mean levels 62.0 (SD 31.5), within normal range | Small study |  |
| Tuokkola 2021  Cross-sectional | Finland | 22 PD, 11 HD | Median 1.2 (IQR 0.8-8.3) yrs | 33 | Supplementation | Fasted red blood cell pyridoxal–5-phosphate | Mean levels above reference range for both on feeds and eating food. Median (IQR) On feeds 22.00 (17.3-40.78) ng/mL, on food 65.87 (35.05-78.40) ng/mL | Small number of children who were not on feeds |  |

**Table S18.** Summary of studies with vitamin B9 dietary intake in children with CKD

| **Author & year, Titles and journal, type of study** | **Location** | **Patients** | **Age** | **N** | **Intervention** | **Outcome measures** | **Findings** | **Potential bias / limitations** | **Notes** |
| --- | --- | --- | --- | --- | --- | --- | --- | --- | --- |
| Coleman JE 1992  Cohort | UK | PD | mean 6.1, Range: 0.3 - 12.6 yrs | 7 | Trial of two supplements for 3 months (750 ug x 3 or 53 ug folic acid) | 3-day food records | Mean intake from diet ~250% RDA, with Ketovite 1256%, with Seravit ~300% | Small study |  |
| Foreman JW 1996, prospective | USA | GFR 20-75 | range 1.5-11.9 yrs, mean 6.1+- 3.2 yrs | 120 children, 401 food records |  | 4-day Food records at enrollment and at 2, 4, and 6 months | Mean B9 intake was low in all except 1-3-yr-olds |  | Well controlled fod records with scales and measuring cups provided for families. |
| Kim H et al 2014  Cross-sectional | South Koreal | CKD 2-5 | 10-19 yrs | 19, 10 GFR > 75 mL/min , 9 < 75 mL/min |  | Dietary intake, 3-day food records | Intake % of RDA in GFR ≥ 75 50+-10%, in GFR < 75 30+-10% |  | Dietary recommenedations intakes for Korea . Ad libitum diet, ht z scores -0.2 +/- 0.8 , wt z score -0.2 +/- 1.3 |
| Kriley & Warady 1991  Cross-sectional | USA | APD, CAPD | 5.3 yrs | 8 patients + 6 healthy controls | Daily supplementation 0.8 mg/day | Dietary intake, 3-day weighed food records | Intake % of RDA 230% controls, 169% diet, 1072% diet + suppl* | Small study. Diet not described (use of sip feeds?) |  |
| Pereira AM 2000  Prospective | Brazil | 4 IPD, 19 CAPD, 7 HD | 9.3 +- 7.4 yrs | 30 | Supplementation | Dietary and supplemental intake adequacy, 3-day food records;  anthropometry | Intakes % of RDA diet PD 136+-79, HD 90+-37, with suppl PD 1456+-1045, HD 1090+-425 | No blood levels |  |
| Warady B 1994  Cross-sectional | USA | PD | 11.4+-2.6 mo | 7 | Supplementation 0.1 mg/day | Dietary intake 3-day food records | Intake from diet 230% of RDA, diet + suppl 585% | Small study |  |
| Tuokkola 2021  Cross-sectional | Finland | 22 PD, 11 HD | Median 1.2 (IQR 0.8-8.3) yrs | 33 | Supplementation | Dietary intake 3-day food records | Intake on feeds 213% of RNI, feeds + suppl 284% food 100%, food + suppl 600% | Small number of children who were not on feeds |  |

**Table S19.** Summary of studies with vitamin B9 biochemical status in children with CKD

| **Author & year, Titles and journal, type of study** | **Location** | **Patients** | **Age** | **N** | **Intervention** | **Outcome measures** | **Findings** | **Potential bias / limitations** | **Notes** |
| --- | --- | --- | --- | --- | --- | --- | --- | --- | --- |
| Canepa A 2003, cross-sectional | Italy | Mean GFR 37.3±16.9 ml/min per 1.73 m2 | mean 10.3±4.7 yrs | 42 CRF, 33 controls |  | Serum B9 | B9 deficiency in 14%, B9 levels lower in subjects than in controls | No information on diet | the predictors of tHcy serum concentrations, folic and vitamin B12 were significant in controls, whereas only GFR was significant in CRF children. 40% of patients with hyperhomocysteinemia |
| Coleman JE 1992  Cohort | UK | PD | mean 6.1, Range: 0.3 - 12.6 yrs | 7 | Trial of two supplements for 3 months (250 ug x 3 or 53 ug folic acid) | Red cell B9 | Ketovite 1011+-293 (785-1470), Paediatric renal seravit 7081+-183 (500-1185) | Small study |  |
| Coleman JE 2002 | UK | GFR < 25, HD, PD | mean 10.4 yrs (range 1.1-16 yrs) | 11 GFR < 25, 2 HD, 2 PD | Comparison of two micronutrient supplements (250-750 ug vs 200-400 ug/day, depending on age) | Serum and red cell B9 | Serum B9 28.8 (12) vs 24 (9.5) ug/L, red cell B9 1435 (526) vs 1136 (427) ug/L |  |  |
| Feinstein S et al 2002, cross-sectional | Israel | PD, HD | 12.3±1.1 | 29 | Supplementation with B9 1.25–2.5 mg/day | red blood cell (RBC) B9, | RBC B9 393±37 ng/ml in unsupplemented, 1211±128 ng/ml in supplemented; all within or above reference range |  |  |
| Joyce T 2018b  retrospective | UK | 28 HD, 19 PD | median 11.4 (2.8, 14.4) | 47 | 12/47 received oral nutritional supplement or enteral feed |  | 39% had high B9 levels |  |  |
| Joyce T 2020  retrospective | UK | CKD2-5 | median 8.97 (IQR 4.24, 13.80) | 112 | diet alone, enteral tube feeding, oral nutritional supplements |  | Diet alone: 5% above reference range, tube fed: 5% above reference range, ONS: within reference range. In all, 5% above, 3% below, 92% within reference range. | No information on dietary intake |  |
| Kriley & Warady 1991  Cross-sectional | USA | APD, CAPD | 5.3 yrs | 8 patients + 6 healthy controls | Daily supplementation 0.8 mg/day | Serum folic acid | Levels higher in patients than controls (60 vs. 19) | Small study. Diet not described (use of sip feeds?) |  |
| Litwin M 2001, cross-sectional | Poland | GFR <20, GFR >20, HD, controls |  | 9 GFR <20, 9 GFR >20, 14 HD, 16 controls | mean ages of groups 18, 18 and 16 yrs, controls 13.5 yrs | relation between plasma sulphur amino acid and folic acid and B12 | Blood Folic acid concentrations higher in patients than in controls | No information on diet | no correlation between plasma AA concentrations and serum B9 and vitamin B12 |
| Warady B 1994  Cross-sectional | USA | PD | 11.4+-2.6 mo | 7 | Supplementation 0.1 mg/day | Serum folic acid | >45 (above reference and mesurement range) | Small study |  |
| Tuokkola 2021  Cross-sectional | Finland | 22 PD, 11 HD | Median 1.2 (IQR 0.8-8.3) yrs | 33 | Supplementation | Fasted serum B9 | Mean levels above reference range for both on feeds and eating food. Median (IQR) On feeds 40.8 (34.1-45.0) nmol/mL, on food 39.4 (19.5-45.0) nmol/mL | Small number of children who were not on feeds |  |

**Table S20.** Summary of studies with vitamin B12 dietary intake in children with CKD

| **Author & year, Titles and journal, type of study** | **Location** | **Patients** | **Age** | **N** | **Intervention** | **Outcome measures** | **Findings** | **Potential bias / limitations** | **Notes** |
| --- | --- | --- | --- | --- | --- | --- | --- | --- | --- |
| Coleman JE 1992  Cohort | UK | PD | mean 6.1, Range: 0.3 - 12.6 yrs | 7 | Trial of two supplements for 3 months (1.5 µg vs 0 µg) | 3-day food records | Mean intake from diet ~250% RDA, with Ketovite ~250%, with Seravit ~400% | Small study |  |
| Don T 2010  Cross-sectional | New Zeeland | PD | 7.8 +-5.3 yrs | 12 | 1 patient with 4 mg supplementation | Dietary intake 3-day food records | total median 313% of DRI (range 108-842%), diet alone 92% | Small study. |  |
| Kriley & Warady 1991  Cross-sectional | USA | APD, CAPD | 5.3 yrs | 8 patients + 6 healthy controls | Daily supplementation 6 µg/day | Dietary intake, 3-day weighed food records | Intake % of RDA260% controls, 210% diet, 735% diet + suppl* | Small study. Diet not described (use of sip feeds?) |  |
| My Thuc LT 2019  Cross-sectional | Vietnam | PD | 8.5 +- 4.2 yrs | 31 |  | Dietary intake, 24-h dietary questionnaire | Intake 166.7% of DRI | 35.5% of patients malnourished |  |
| Tuokkola 2021  Cross-sectional | Finland | 22 PD, 11 HD | Median 1.2 (IQR 0.8-8.3) yrs | 33 | Supplementation | Dietary intake 3-day food records | Intake on feeds 300% of RNI, feeds + suppl 338% food 195%, food + suppl 420% | Small number of children who were not on feeds |  |
| Warady B 1994  Cross-sectional | USA | PD | 11.4+-2.6 mo | 7 | Supplementation 2.5 µg/day | Dietary intake 3-day food records | Intake from Diet 273% of RDA, diet + suppl 752% | Small study |  |

**Table S21.** Summary of studies with vitamin B12 biochemical status in children with CKD

| **Author & year, Titles and journal, type of study** | **Location** | **Patients** | **Age** | **N** | **Intervention** | **Outcome measures** | **Findings** | **Potential bias / limitations** | **Notes** |
| --- | --- | --- | --- | --- | --- | --- | --- | --- | --- |
| Canepa A 2003, cross-sectional | Italy | Mean GFR 37.3±16.9 ml/min per 1.73 m2 | mean 10.3±4.7 yrs | 42 CRF, 33 controls |  | Serum B12 | VitB12 deficiency in 5%. B12 levels higher in subjects than in controls | No information on diet | the predictors of tHcy serum concentrations, folic and vitamin B12 were significant in controls, whereas only GFR was significant in CRF children. 40% of patients with hyperhomocysteinemia |
| Coleman JE 1992  Cohort | UK | PD | mean 6.1, Range: 0.3 - 12.6 yrs | 7 | Trial of two supplements for 3 months (1.5 µg vs 0 µg) | Serum cobalamin | Mean levels above normal range in both groups. Ketovite 990+-358 (410-1310), Paediatric renal seravit 1132+-378 (660-1560) | Small study |  |
| Coleman JE 2002 | UK | GFR < 25, HD, PD | mean 10.4 yrs (range 1.1-16 yrs) | 11 GFR < 25, 2 HD, 2 PD | Comparison of two micronutrient supplements (0 vs. 0.5-1.0 µg/day, depending on age) | Serum B12 | Serum B12 556(385) vs 578 (332) ng/L (reference range 300-1100) | Small study |  |
| Don T 2010  Cross-sectional | New Zeeland | PD | 7.8 +-5.3 yrs | 12 |  | Serum B12 | Mean levels above reference range | Small study. | No correlation between dietary  intake and the biochemical status |
| Feinstein S et al 2002, cross-sectional | Israel | PD, HD | 12.3±1.1 | 29 | - | plasma Hcy, red blood cell (RBC) B9, and serum vitamin B12 | Blood B12 concentrations 488±40 pg/ml; in the normal range for all patients |  |  |
| Litwin M 2001, cross-sectional | Poland | GFR <20, GFR >20, HD, controls | mean ages of groups 18, 18 and 16 yrs, controls 13.5 yrs | 9 GFR <20, 9 GFR >20, 14 HD, 16 controls |  | relation between plasma sulphur amino acid and folic acid and B12 | No difference in blood B12 concentrations between patients and controls, levels within reference ranges | No information on diet | no correlation between plasma AA concentrations and serum B9 and vitamin B12 |
| Joyce T 2018b  retrospective | UK | 28 HD, 19 PD | median 11.4 (2.8, 14.4) yrs | 47 | 12/47 received oral nutritional supplement or enteral feed |  | Blood levels of vitB12 20% normal, 80% high | No information on diet |  |
| Joyce T 2020  retrospective | UK | CKD23-5 | median 8.97 (IQR 4.24, 13.80) yrs | 112 | diet alone, enteral tube feeding, oral nutritional supplements |  | Diet alone: 29% within and 72% above reference range, tube fed: 5%within and 95% above reference range, ONS: within reference range. In all, 77% above, 23% within reference range. | No information on diet |  |
| Kriley & Warady 1991  Cross-sectional | USA | APD, CAPD | 5.3 yrs | 8 patients + 6 healthy controls | Daily supplementation 6 µg/day | Serum B12 | NS difference between controls and patients | Small study. Diet not described (use of sip feeds?) |  |
| Tuokkola 2021  Cross-sectional | Finland | 22 PD, 11 HD | Median 1.2 (IQR 0.8-8.3) yrs | 33 | Supplementation | Serum B12 | Mean levels above reference range for both on feeds and eating food. Median (IQR) on feeds 862 (637-1096) pmol/mL, on food 606.5 (254.8-761.5) pmol/mL | Small number of children who were not on feeds |  |
| Warady B 1994  Cross-sectional | USA | PD | 11.4+-2.6 mo | 7 | Supplementation 2.5 µg/day | Serum B12 | Mean (SD)1016.3 (199.2), reference range 89-590 | Small study |  |

**Table S22.** Summary of studies with vitamin C dietary intake in children with CKD

| **Author & year, Titles and journal, type of study** | **Location** | **Patients** | **Age** | **N** | **Intervention** | **Outcome measures** | **Findings** | **Potential bias / limitations** | **Notes** |
| --- | --- | --- | --- | --- | --- | --- | --- | --- | --- |
| Foreman JW 1996, prospective | USA | GFR 20-75 | range 1.5-11.9 yrs, mean 6.1+- 3.2 yrs | 120 children, 401 food records |  | 4-day Food records at enrollment and at 2, 4, and 6 months | Mean vitC intake normal in all age groups |  | Well controlled food records with scales and measuring cups provided for families. |
| Hongsawong Nattaphorn 2021, prospective | Thailand | 7 CKD 4,  12 PD | 12 +/- 4.10 yrs | 19 | oral Vit C supplementation of 250mg/d for 3 months in those with deficiency or insuffiency as indicated by serum vitamin C | 24-h recall | Dietary intake 36.59 ( 0.0-63.81) % RDI  CKD stage 4 n=7 22.65( 5.12-26.50) mg/d ,  CKD stage 5d n= 12 11.4 ( 0-36.25) mg/d  total n= 19 17.10 ( 0-26.52) mg/d  serum Vit C sig increased with supplementation without sig increase of serum oxalate. However almost 2/3 failed to reach normal serum levels of vit C. Dose of epo reduced after receiving Vit C but not statistically significant. For Iron and anemia status no sig change | only PD pts and CKD 4 no HD. 24hr dietary recall as assessment of intake , shown in some of the results 1 pt had high intake and serum insufficiency, and those with normal vit c level (2) had low intakes. Small numbers. Short follow up |  |
| Kriley & Warady 1991  Cross-sectional | USA | APD, CAPD | 5.3 yrs | 8 patients + 6 healthy controls | Daily supplementation 60 mg/day | Serum ascorbic acid, dialysate ascorbic acid | Dietary intake 199% controls, 131% diet, 259% diet + suppl, NS  losses in dialysate 32 mg/d |  |  |
| My Thuc LT 2019  Cross-sectional | Vietnam | PD | 8.5 +- 4.2 yrs | 31 |  | Dietary intake, 24-h dietary questionnaire | Intake 133.7% of DRI | 35.5% of patients malnourished |  |
| Pereira AM 2000  Prospective | Brazil | 4 IPD, 19 CAPD, 7 HD | 9.3 +- 7.4 yrs | 30 | No vitamin C supplementation | Dietary and supplemental intake adequacy, 3-day food records;  anthropometry | Intakes % of RDA diet median 67%; in PD, 77%+-47, in HD, 51%+-32 | No blood levels |  |
| Tuokkola 2021  Cross-sectional | Finland | 22 PD, 11 HD | Median 1.2 (IQR 0.8-8.3) yrs | 33 | Supplementation in those not on feeds | Dietary intake 3-day food records | Intake on feeds 644% of RNI, food 135%, food + suppl 283% | Small number of children who were not on feeds |  |
| Warady B 1994  Cross-sectional | USA | PD | 11.4+-2.6 mo | 7 | Supplementation 15 mg/day | Dietary intake 3-day food records | Intake from diet 140% of RDA, diet + suppl 182% | Small study |  |

**Table S23.** Summary of studies with vitamin C biochemical status in children with CKD

| **Author & year, Titles and journal, type of study** | **Location** | **Patients** | **Age** | **N** | **Intervention** | **Outcome measures** | **Findings** | **Potential bias / limitations** | **Notes** |
| --- | --- | --- | --- | --- | --- | --- | --- | --- | --- |
| Hongsawong Nattaphorn 2021, prospective | Thailand | 7 CKD 4 12 PD | 12 +/- 4.10 yrs | 19 | oral Vit C supplementation of 250mg/d for 3 months in those with deficiency or insuffiency as indicated by serum vitamin C | Serum vit C and oxalate, anemia status | serum Vit C sig increased with supplementation without sig increase of serum oxalate. However almost 2/3 failed to reach normal serum levels of vit C. Dose of erythropoietin reduced after receiving Vit C but not statistically significant. For Iron and anemia status no sig change |  |  |
| Naseri M 2015, prospective | Iran | 12 CAPD, 25 HD | 19-300 mo, (166 +/- 76 mo) | 43 (37 for vit C) | Supplementation in 18/43 patients, 30–60 (51.2) mg/day | Serum vitC | Vit C ( n 37) low in 13.5 %, normal 86%. all cases of vit C def were in HD pts ( sig) , all cases with combined vit c and e def were HD pts . HD pts in vit C grp n= 23 , 5 had a low vit C level , CAPD grp(n =9) none had low vit C level - 7 of these were supplemented . 18 pts in total supp with vit C all had normal vit C. Pt s that stopped vit C ( n= 11) supp 2 had low serum level vit C depleation is sig , | vit C supp recommeended, half grp stopped or used irregularly . | last results showed not sig difference in serum hb in pts with vit C def or not. |
| Tuokkola 2021  Cross-sectional | Finland | 22 PD, 11 HD | Median 1.2 (IQR 0.8-8.3) yrs | 33 (vit C 12) | Supplementation | fasted plasma ascorbic acid | Median blood levels within range, 15.4% of patients below normal range. Median (IQR) on feeds 13.0 (5.2-22.0) mg/L, on food 0.5-1.0 mg/L, reference range 2-20 | Small number of children who were not on feeds |  |
| Warady B 1994  Cross-sectional | USA | PD | 11.4+-2.6 mo | 7 | Supplementation 15 mg/day | Serum ascorbic acid | Mean (SD) 116.7 (44.6), reference range 22.8-113.8. None below reference range. | Small study |  |
| Zwolinska et al, 2006, cohort | Poland | 21 HD, 27 controls | Mean 12.9 yrs | 48 | HD patients received 100mg daily of vit C, duration not clear | Plasma and dialysate vitC. All but HD children had fasted samples. Analaysis of vit C by HPLC. | low levels seen across all groups ( 2 grps in the study with CKD but not on dialysis), lowest levels in those on HD. Large amount of vitC in HD dialysis fluid (0.415+-0.113 µmol/L). (=73.04 +-19.89 mg/L) | Vitamin A concentrations are low - questions validity of study as not typical of CKD. |  |
| Zwolinska et al 2009, cross-sectional | Poland | PD | 13.2+-3.5 yrs | 10 PD + 27 controls | Mention that supplementation is usually restricted to 60-100 mg/day | Fasted plasma and dialysate concentrations | Significant vitamin C losses in dialysate: plasma in controls 105.77+-6.07, in PD patients 88.4+-4.9, in dialysate 1.36% 4.48+-0.54µmol/L, in dialysate 2.27% 4.66+-0.45µmol/L | No information on diet or supplementation |  |

**Table S24.** Summary of studies with vitamin B9 and B12 interventions in children with CKD

| **Author & year, Titles and journal, type of study** | **Location** | **Patients** | **Age** | **N** | **Intervention** | **Outcome measures** | **Findings** | **Potential bias / limitations** | **Notes** |
| --- | --- | --- | --- | --- | --- | --- | --- | --- | --- |
| Bamgbola 2005, cross-over intervention | USA | HD | 16+-3.5 yrs | 15 | unsupplemented 6 mo, supplemented 5 mg 3 x per week for 6 mo | Serum and RBC B9s, serum B12, tHcy, EPO resistance | Hb increased 8%, reduction in MCV and RDW. S-fol increased by 38%, RBC by 24%. 26% B9 deficient. EPO dose decreased with B9 supplementation. |  |  |
| Bennett-Richards K 2002, DBPC cross-over intervention | UK | CRF, GFR 26.8+-13.2, normotensive | 12 +- 3 yrs | 25 | Folic acid 5 mg/m2, 8 weeks + 8 wk washout | HCy levels, LDL oxidation, vascular function | serum B9 levels rose  from 11.74.25 to 635519 g/L (P0.001), red cell B9 levels rose from 364195 to 28912623 g/L (=P0.001). Lag times for LDL oxidation were prolonged during the treatment  phase (58.418.7 to 68.125.9 minutes, P0.01). improvement in flow-mediated dilatation (FMD) (endothelial-dependent dilatation) from 7.212.8% to  8.473.01% (P0.036)  At entry to the study, serum B9  (13.73.58 g/L) and red cell B9 levels (334202 g/L)  were normal. Folic acid produced a significant increase in  both serum B9 (11.74.25 to 635519 g/L, P0.001)  and red cell B9 (364195 to 28912623 g/L, P0.001)  levels during the treatment period. |  |  |
| Coleman JE 1992  Cohort | UK | PD | mean 6.1, Range: 0.3 - 12.6 yrs | 7 | Trial of two supplements for 3 months (750 ug x 3 or 53 ug folic acid) | Red cell B9 | Ketovite 1011+-293 (785-1470), Paediatric renal seravit 7081+-183 (500-1185) | Small study |  |
| Coleman JE 2002 | UK | GFR < 25, HD, PD | mean 10.4 yrs (range 1.1-16 yrs) | 11 GFR < 25, 2 HD, 2 PD | Comparison of two micronutrient supplements (250-750 ug vs 200-400 ug/day, depending on age) | Serum and red cell B9 | Serum B9 28.8 (12) vs 24 (9.5) ug/L, red cell B9 1435 (526) vs 1136 (427) ug/L |  |  |
| Schröder CH 1999  open trial | Netherlands | HD, PD | HD 10.6+-4.1, PD 8.5+-4.5 yrs | 21 (9 HD, 12 PD), 234 healthy controls | Folic aid supplement 2.5 mg / day | Plasma folic acid and HCy levels aseline and after supplementation | HCy decreased from 20.0 μmol/l to 12.0 μmol/l with supplementation.  Folic acid concentrations within reference range before supplementation |  |  |

**Table S25.** Summary of studies with vitamin C interventions in children with CKD

| **Author & year, Titles and journal, type of study** | **Location** | **Patients** | **Age** | **N** | **Intervention** | **Outcome measures** | **Findings** | **Potential bias / limitations** | **Notes** |
| --- | --- | --- | --- | --- | --- | --- | --- | --- | --- |
| El Mashad GM 2016  RCT | Egypt | HD | in supplementation grp 8.2 +/- 17.3, in placebo 9.5 +/- 3.1 yrs | 30 suppl + 30 placebo | IV vit C 250mg post HD session x3 times a week | serum uric acid, plasma ascorbic acid , serum TG and serum chol , HDL and LDL . Fasting samples were taken pre HD and after 3 months of the study. | at the end of the study period vit C levels increased sig and serum uric acid decreased sig in supplemented grp and sig reduction in the levels of lipids ( trig, LDL, and chol) , increase in HDL | small number , short duration. Oxalate not measured |  |
| Kennedy S 2020, retrospective | US | PD, HD? | 42 mo sd 24 (18 mo - 5.5 yrs) | 13 | all formula fed, 12 on vit c supp. 13 receiving 145-847% of DRI | serum ascorbic acid and plasma oxalate | mean levels of AA and oxalate were elevated | small numbers, no association found between vit c intake and aa and oxalate levels |  |

**Table S26.** Summary of studies with vitamin B complex in children post kidney transplant

| **Author & year, Titles and journal, type of study** | **Location** | **Vitamins studied** | **Age** | **N** | **Intervention** | **Outcome measures** | **Findings** | **Potential bias / limitations** | **Notes** |
| --- | --- | --- | --- | --- | --- | --- | --- | --- | --- |
| Harmer M et al 2019, observational | UK | B6 | mean 11.9 yrs | 10 | - | 1. dietary assessment 2. serum Pyridoxal 5'-phosphate (PLP) measurement | 1. Mean vitamin B6 intake was 138.7% of reference nutrient intake (standard deviation ±35.2%) 2. median serum PLP concentrations of 62.45 nmol/L (interquartile range ±83.40). 3. no children appeared biochemically deficient, but 20% had elevated concentrations 4. Dietary intakes were not excessive, and no children reported oral Vitamin B6 supplementation |  |  |
| Hamatani R et al 2014, cross-sectional | Japan | B9 | age at RTx 12.6 ± 4.1 yrs; age during study: 21.2 ± 5.5 yrs | 89 | - | plasma homocysteine and plasmja B9 | 1. 60 (67.4 %) had hyper-Hcy and 14 (15.7 %) had plasma B9 deficiency. 2. Plasma homocysteine levels correlated negatively plasma B9 levels (r = -0.434, p < 0.01) 3. Dietary B9 intake in 11 of 16 patients (66.8 %) with eGFR ≥ 60 ml/min/1.73 m(2) was below the recommended dietary allowance for Japanese, 3.No patients were on folic acid supplementation |  |  |
| Aldámiz-Echevarría L et al 2002, cross-sectional | Spain | B9, B12 | 2-18 yrs | 26 | - | Plasma levels | 1. Plasma concentrations of folic acid and vitamin B12 were all within normal range, 2.Plasma tHcys correlated negatively with creatinine clearance ( r=-0.58, P<0.001) and plasma vitamin B(12) ( r=-0.40, P<0.05) but no with folic acid |  |  |
| Merouani A et al 2002, prospective | Canada | B9, B12 | mean age at the time of RT of 10.3±4.8 yrs (range 5–17 yrs) | 6 | folic acid supplementation was introduced after the evaluation at 6 months | Plasma homocysteine, vitamin B(12), and B9 concentrations | 1. No significant changes were observed in B9, and vitamin B(12) concentrations before at 6 months post RT. 2. Median B9 Z scores were 1.89 during dialysis, –0.26 at 6 months, and 3.26 at 4 years post RT. Median vitamin B12 Z score was 2.12 during dialysis, 0.58 at 6 months, and –0.07 at 4 years post RT.3. Elevated plasma homocysteine in children during dialysis persists after RT despite a significant improvement in renal function. 3. Normalization was attained when patients were supplemented with folic acid. |  |  |
| Feinstein S et al 2002, cross-sectional | Israel | B9, B12 | 12.3±1.1 yrs | 34 | - | plasma Hcy, red blood cell (RBC) B9, and serum vitamin B12 | Following Tx, both serum vitamin B12 and RBC B9 levels substantially decreased, although they remained within normal limits regardless of the individual’s renal function |  |  |
| Pontes KSDS et al 2019, cross-sectional | Brazil | B12 | 4.50 (sd 12·11) yrs | 225 |  | 1. Plasma levels, 2. three 24 h dietary recalls | B12 deficiency in KTR was estimated as 14 % and was associated with reduced intake of B12 as well as higher adiposity, especially in women, and with the use of MMF |  |  |

**Table S27.** Non-diet causes of abnormal status: accumulation, losses, effects of medication: Fat soluble vitamins A, E, K

| **Search 1980 to 2021** | |  |  |  |  |  |  |  |  |  |
| --- | --- | --- | --- | --- | --- | --- | --- | --- | --- | --- |
| **Author & year, type of study** | **Location** | **Vitamin(s)** | **Patients** | **Age** | **N** | **Intervention** | **Outcome measures** | **Findings** | **Potential bias / limitations** | **Notes** |
| Werb 1979 | USA | A | Adult dialysis | 19-72 | 72 | Evaluation of vitamin A in dialysis patients vs healthy controls | Serum levels evaluated pre-dialysis and post-dialysis | Dialysis patients had significantly higher vitamin A levels compared to controls; those with supplement vitamin A had highest levels | Minimal lab data available | vitamin A levels did not decrease post-dialysis compared to pre-dialysis.  1st study to identify that vitamin A was not excreted through dialysis |
| Manickavasagar, 2015 | UK | A | Pediatric CKD and dialysis | 3-17 | 105 | Evaluation of vitamin A levels compared to healthy controls; evaluation of dietary intake | Serum levels and dietary intakes | Elevated vitamin A levels even for patients with intakes below DRI |  |  |
| Blumberg 1983 | USA | A | Adult PD patients | adults | 10 | Evaluation of vitamin A levels before and after supplementation | Serum levels | Elevated vitamin A levels before and after supplementation | Limited specific data |  |
| Hultqvist et al, 1997 | Sweden | E | Adult HD patients | Adults | 13 | HD | Dialysis losses | No alpha-tocopherol was detected in the dialysate | Limited numbers. | No change in plasma concentrations of alpha-tocopherol pre- / post-HD, and none lost in dialysate. |

**Table S28.** Non-diet causes of abnormal status: accumulation, losses, effects of medication: Water soluble vitamins C and B complex

| **Search 1980 to 2021** | |  |  |  |  |  |  |  |  |  |
| --- | --- | --- | --- | --- | --- | --- | --- | --- | --- | --- |
| **Author, year, type of study** | **Location** | **Vitamin(s)** | **Patients** | **Age** | **N** | **Intervention** | **Outcome measures** | **Findings** | **Potential bias / limitations** | **Notes** |
| Morena M 2002 [[1]](https://www.zotero.org/google-docs/?LHShLE)  Cross-sectional | France | C | Adult HD | 62+/- 7 yrs | 19 | Evaluation of vitamin C in dialysis patients vs healthy controls | Serum samples taken pre and post HD and at 60 mins.  At 60 mins UF and dialysate samples also taken | Significant reduction in vitamin C levels vs healthy controls  1.87+/- 1.57 to 0.98+/-0.68  Total loss of vitamin C in dialysate 66 mg/session (8-230mg) | Not clear if fasted samples | The higher the vitamin C level at the beginning of the session the greater the loss  Not on vitamin C supplement |
| Zwolinska D  2006 [[2]](https://www.zotero.org/google-docs/?0Ey70t)  Cross-sectional | Poland | C | Pediatric HD | 11 to 16 | 21 on HD  46 pre dialysis | HD pts on 100mg daily vitamin C supplement | Serum vitamin C pre HD, fasted sample for pre dialysis group, dialysate samples | Plasma concentration of vitamin C significantly low in all groups.Lowest level in HD group.  Large amount of vitamin C in dialysate micromols/l 0.415 +/-0.113 | Non fasted samples for HD pts |  |
| Bohm V 1997 [[3]](https://www.zotero.org/google-docs/?a6c2jo)  Cross-sectional | Germany | C | Adults CKD,,HD,  post transplant | 21-85 | 17 HD,  23 CKD,  22 post transplant | HD group 60 or 100 mg vit C supp x3 a week after HD session for 14 days | Fasted serum samples at day 7, day 14, post HD and 44h after HD. Urine (were possible) and dialysate.(n=3)  Dietary intake | Mean decrease in concentration of plasma vitamin C 50%. Total loss of vitamin C in dialysate 93-334mg over a 4 hr session.  44 hr after HD the plasma vitamin C reached 90-98% of the initial value regardless of vitamin supplementation and dietary intake |  |  |
| Fehrman-Ekholm I 2008. [[4]](https://www.zotero.org/google-docs/?yGCmGE)  Cross-sectional | Sweden | C,B9,B12, | Adult HD | Adults | 19 | Low flux vs conventional HD | Serum pre and post dialysis  at mid week session. | Vitamin C significantly reduced in both groups 51% and 53%.  12/14 not on supplementation had vitamin C below the reference value.  1 on 200mg had levels below reference value  B9 and B12 not reduced  10 on B9 supplementation  11 on B12 supp  all had B12 above reference value |  |  |
| Coveney N 2011 [[5]](https://www.zotero.org/google-docs/?KypPR4)  Cross-sectional | Australia | C B1,B6, B9 B12. | Adults | HD | 52 | Extended vs conventional HD  Supplement B1 7.27 mg, B2 6 mg,  B3 45 mg, B6 HCl 0.7mg, C 45 mg / day B9 5 mg/week. Extended group used more supplements than conventional | Serum pre and post HD | Significantly lower vitamin C in extended hrs grp - 72% def. No deficiency in conventional group  Vit C serum mean values 0.3/dL mcg in extended, 1.14mcg/dL in conventional HD. Levels were lower as the number of weekly hours on dialysis increased.  B9 no deficiency and no significant difference in values between groups.  B6 extended group had higher levels and none were deficient. Conventional group higher incidence of deficiency ( n=9 34%) - not on supplement.  B1 no deficiency in extended group lower mean values then conventional group- 1 deficient in conventional group (on supplementation and known to have malnutrition).  B12 1 in conventional group deficient no significant difference between 2 groups re mean values. |  | Comparison of duration of weekly  Conventional group on dialysis longer and older. |
| Blumberg A 1983 [[6]](https://www.zotero.org/google-docs/?6yZBXw)  Trial | Switzerland | A,E,B complex  and C | Peritoneal dialysis (PD) | Adults | 10 | Supplementation B1( 8mg),B2 (8mg),B6(10mg),B3 (50mg),B5 (10.9 mg),B9 ( 2mg) and C(100mg) | Serum B1, B2, B6, B9, A,E,C, taken pre supplementation,7 and 13 weeks post. Dialysate for AE and C. Weighed diet diary. | Vitamin C losses in dialysate 4.8 +/- 1.2 mg/l pre supplementation and  10.5+/- 1.8 mg/l after supplementation. Serum vitamin C normalized on supplementation.  Mean serum levels increased from 7.7mg/l to 16.7 mg/l  Pre supp B1 low in 3 and borderline in 2  B1 stayed low in serum for 5.  B2 sufficient levels pre and post.  B6 pre supplementation 80% deficient.  B6 improvement seen in 90%.  Pre supplementation B9 level low in 4  then went too high in 7.  B12 not supplemented was normal in all slight decline in serum levels after 13 weeks.  Dietary vitamin intake was below the recommended for  B1 - 60% of pts  B2 10%  B3 80%  B6 100%  B12 70%  C 20% |  |  |
| Boeschoten EW 1988 [[7]](https://www.zotero.org/google-docs/?hDRRQY)  Trial | Netherlands | B1,B2,B6,B9,B12,A,E,C, | PD | Adults | 44; | 10 patients daily supplement  B6 2 mg  C 100 mg  B9 400 mg  For 12 weeks | Serum and dialysate ( average of 3 measurements of 36 pts )  Supplemented group bloods taken at weeks 0,1,4,8,12 and 16    Diet diary in 15 | Deficiency seen in B1,B6,B9 and C.  Extremely high loss of vitamin C and B9 in dialysate, negligible for B12  low B1, relatively high B2 and B6  (When compared to urinary loss in a healthy adult ).  losses in dialysate n= 36  C 56 +/- 8mcg  B1 46 +/- 3 mcg  B2 832 +/- 145 mcg  B6 747 +/- 111mcg  B9 107+/- 5mcg  *per session*  losses in urine of healthy adults is >5mg/d  Diet analysis also showed intake below  recommendations;  B6 all intake below recommendations  B1 only 2 had recommended or above intake  B2 same as b1  C 7 had recommended or above intake  intake  B9 not described  Those on a supplementation increase in serum levels within a week.  Vitamin C and B9 went to normal range. 4 weeks after stopping supplement vitamin C serum dropped to pre supplement level.  B9 normal serum levels prior to supplement went to supra normal value, after 4 weeks of stopping supplement levels were still significantly higher. |  | High loss B2 in dialysate but serum level normal even with low reported dietary intake  Serum levels of B9 may be explained by re-circulation in the enterohepatic cycle  B12 binds to serum protein.  similar loss of B6 in dialysate to B2 and deficiency  seen and drop of serum level after stopping supp |
| Finkelstein FO 2011  [[8]](https://www.zotero.org/google-docs/?IowPYg)  Trial | US | C | PD | 57 +/- 16 yrs | 56 | Vitamin C supplement 60-100 mg/d | Serum, dialysate and  3 day diet diary | Serum vitamin C significantly associated with higher Hb levels.  4 of the 9 had more than 40mg of vitamin C in dialysate.  Serum vitamin C  mean 33 +/- 34 median 22 micromol/l. Intake (diary in 23 patients) varied 60-400mg including diet and supplements. |  | Dialysate loss reduced with a lower serum level |
| Shah G 1991 [[9]](https://www.zotero.org/google-docs/?sGLKrc)  Trial | US | C and B6 | 57-74 yrs old | PD | 7 | Phase 1  all vitamins stopped for 4 weeks.  Phase 2  4 weeks of 100mg vitamin C.  Phase 3  addition of B6 10mg for 4 weeks. | Dialysate at last day of each phase.  Serum vitamin C weekly phase one and at the end of phase 2 and 3.  Serum PLP ( precursor of vitamin B6 ) at end of each phase.  3 day diet diary completed on last 3 days of each phase. | Serum vitamin C levels dropped in phase one from 74 +/- 11mol/L to 62 +/- 11 at the end of week 3 ( stayed the same at week 4 ) all within normal limits. At end of phase 2 plasma vitamin C 91 +/- 6 ( p<0.001)  B6 low at end of phase 1 and 2 ( although levels remained the same )  at end of phase 3 B6 significantly increased and all in normal range  Vitamin C at end of phase 3 102 ( slightly raised not significant ).  No difference seen with serum vitamin C between those that were anuric or not.  Dialysate removal of vitamin C was 50mg +/- 6mg/d in phase 1  increased to 79 +/- 6 mg /d by end of phase 2 ( significant) phase 3 90 +/- 9 mg/d. .  Peritoneal clearance  of vitamin C remained unchanged across all 3 phases  Vit C intake across all phases not significantly different 80-113 mg/d +/- 14- 25 mg/d ( 133-200% recommended intake)  B6 dietary intake was suboptimal across all phases. |  | ? 3 weeks to achieve steady state in healthy this may have possibly also been the case in this study |
| Wang, S 1999 [[10]](https://www.zotero.org/google-docs/?CS0ALr)  Cross-sectional | Norway | C | HD | 35-77 yrs old | 19 | 12 dialysis clearance  7 200mg vitamin C supplement for 2 weeks | Serum and dialysate  Pre and post serum sample as well as samples taken from arterial and venous samples of the dialyser 1 hr post HD | Median plasma vitamin C reduction of 33% post HD session. |  | No vitamin C for 1 month prior to the study |
| Kriley & Warady 1991 [[11]](https://www.zotero.org/google-docs/?QhvId4)  Cross-sectional | USA | Vitamin C  APD, CAPD | 5.3 yrs | 8 patients + 6 healthy controls | Daily supplementation 60 mg/day | Serum ascorbic acid, dialysate ascorbic acid | Dietary intake 199% controls, 131% diet, 259% diet + suppl, NS  losses in dialysate 32 mg/d |  |  |  |
| Sirover, W.D 2015 [[12]](https://www.zotero.org/google-docs/?WUZTPm)  Cross-sectional | US | C | HD | Adult | Group 1 = 8  Group 2 = 203 | Group 1 ascertain vitamin C loss in a single session  Group 2 compare those on supplementation vs dietary intake  188 completed diet ass  148 took AA supp | Serum vitamin C | Reduction of 60% vitamin C in serum after a single session of HD. No significant difference on % loss seen depending on pre HD serum level.  Significant difference in serum vitamin C pre HD not on supplement vs those on supplement 37 % had a low vitamin C ( approx half not on a supplement) 70% ( ⅓ not on supp ) had a high pre HD serum level. |  | Dietary assessment - carried out on day of plasma sampling showed a list of 10 common foods and beverages with vitamin C in and asked to report intake of these on the 3 prior days.  48 hrs plasma vitamin C mean not far from pre HD levels |
| Raimann JG 2019 [[13]](https://www.zotero.org/google-docs/?QX5X6X)  Trial | US | C | HD | 50+/- 12 yrs | 44 | 3 vs 6 HD sessions a week | Serum vit C at baseline and months 3,5 and 11 | No significant difference in Vitamin C status on those on more frequent HD |  |  |
| Naseri M 2015 [[14]](https://www.zotero.org/google-docs/?ACI4kY)  Cross-sectional | Iran | C and E | Children HD or PD | 19-300 months , (166 +/- 76 months) | 12 CAPD  25 HD  6 pts during treatment switched RRT | RRT | Serum monthly | Vit C ( n 37) low in 13.5 %, normal 86%. all cases of vitamin C deficiency were in HD pts ( significant) , all cases with combined vitamin C and E deficiency were on HD..Vitamin C deficiency was more prevalent in HD versus CAPD patients (P = 0.128).  Last results show no significant difference in serum Hb in thise with vitamin C deficiency or not.  HD pts in vit C grp n= 23 , 5 had a low serum vitamin C. . CAPD (n =9) none had low serum vitamin C 7 of which were supplemented . 18 pts in total supplemented with vitamin C all had normal serum vitamin C. Those that stopped supplementation C ( n= 11) 2 had low serum level. | Not easy to work out RRT and supplementation status/compliance/dose of the participants, low numbers, other then supplementation no assessment of intake  The 18 that were supplemented none had low serum levels ( cannot work out RRT modality).  Of the 11 that stopped supplementation had low serum levels again ? RRT modality |  |
| Mydlik M 1985 [[15]](https://www.zotero.org/google-docs/?11YEMS)  Cohort | Czechoslovakia | C, B1, B2, B6 | PD | Mean 42 yrs | 10 | Vitamin supplementation  B1 2 mg  B2 2 mg  B6 6 mg  C 200 mg  oral daily | Serum  and dialysate ( just for C )  3 month intervals for 1 yr | Losses of C in dialysate 149 mcmol/6 hrs results from another paper by the same author (1983).  Serum vitamin C was significantly increased with the highest level at onset, stabilized and still significantly higher than the control group.  B1 effect on enzyme significantly increased at onset 3 months to 12 months stayed in range of the control group.  B2 - enzyme activity  significantly decreased as compared to the control group.  B6- range of control of all through study. |  | B1,B2 and B6 determined by indirect method ie assessing catalytic enzyme activity  Not clear when supp started |
| Henderson I 1984 [[16]](https://www.zotero.org/google-docs/?a5OJik)  Trial | UK | A, E B1,B2, B6,B9 B12, C | PD | mean age 36 ( 22-63) | 9 | Vitamin supp =  daily  B9 5 mg  B1 50 mg  B2 6 mg  B6 50 mg  B3 210mg  C 100 mg | Serum pre starting PD , 6 months post PD and vitamin supplementation At 6 months vitamin supplement stopped and serum checked again 6 months later. | Serum vitamin C ULN pre starting PD and did not change at 6 months after starting PD and supplementation. After 6 months with no supplement serum levels significantly decreased to the lower limit of normal.  B1,B2,B6 status pre PD was within normal range and showed no significant change after 6 months PD and supp  6 months after stopping supplement B1 and B2 status remained unchanged but B6 dropped sig although mean value was still within normal rang.  B9 assays difficult as have a ceiling above which accurate quantities were not possible.  Pre PD and not supplemented all levels above ULN and many over the upper limit of assay. After 6 months of PD and supplement all above the upper limit of assay. After 6 months of no supplement all levels fell significantly but were still above the ULN. |  | not on a potassium restriction pre starting PD |
| Marumo F 1986 [[17]](https://www.zotero.org/google-docs/?PuYDq6)  Cross-sectional | Japan | B1,B2,B6, B9, B12,C, | HD and  CKD | 22- 76 yrs | 35 on HD  15 early CKD, 14 advance CKD,36 healthy controls | Evaluation of vitamin status in HD,CKD vs healthy controls. | Serum | No difference in serum vitamin C between control and HD.  Lowest serum vitamin C level in the early CKD group.  Serum B1 significantly lower in HD ( whole blood conc not enzyme activity)  B2 in HD group significantly elevated and same for ureamic vs healthy controls.  B6 significantly higher in HD vs controls.  B12 significantly higher in uremic and HD.  B9 same as B12 |  | The author discusses intake of vitamin C foods higher in Japanese population and differences in food preparation.  Slight K restriction in the non HD groups  Potassium restriction in the HD group. |
| Descombes E 1993 [[18]](https://www.zotero.org/google-docs/?IZUQ8E)  Cohort | Switzerland | C , B1,B2, B6,B7 B9, B12 | HD | 59 +/- 13 yrs | 43 | Pre-study low dose vitamin C ( 200mg post HD session) and B9.  supplementation given depending on result of baseline data  (1 mg post HD session ) | Serum | No systematic supplementation needed for B12, B3,B7  Vit C and B9 previous low dose supp allowed for optimal level in the majority.  Severe B6 deficiency was seen in > 80 % of un supplemented. a post HD supp of 80-120mg a week rapidly normalized serum levels in majority of pts ( 40mg 2-3x a week post HD) more supplement needed depending which enzyme measured 300-450mg a week.  B1 - levels of enzyme low but whole body levels normal - suggests impaired metabolism rather than true deficiency . Supplementation normalized serum enzyme activity levels - varying dose and duration to normalize the 2 different enzymes. |  |  |
| Canavese C 2005 [[19]](https://www.zotero.org/google-docs/?RJUXJ4)  Trial | Italy | C | HD | Adults 3 | 30 | 250mg intravenous vitamin C increased to 500 mg once per week over 18 months. | Serum oxalate and vitamin C | Vitamin C increased from baseline 1.6 to 2.8 and then 6.6 mg/L. Oxalate increased from baseline 35 to 39 and then 50 mcmol/L.  The [calcium oxalate](https://www.sciencedirect.com/topics/medicine-and-dentistry/calcium-oxalate) saturation threshold was exceeded by 7 of 18 patients (40%*)* during 6 months therapy with 500 mg/wk. Vitamin C dialysis removal increased from 37.8 ± 23.2 mg (215 ± 132 μmol) to 99.6 ± 51.7 mg (566 ± 294 μmol) during supplementation (P < 0.001), with corresponding increases in oxalate removal from 82.5 ± 33.2 mg (917 ± 369 μmol) to 111.2 ± 32.6 mg/L (1,236 ± 362 μmol; P < 0.01). Withdrawal reverted plasma levels and dialysis removal to initial values. |  |  |
| Mydlik, M 1999 [[20]](https://www.zotero.org/google-docs/?T9WAY3)  Trial | Slovakia | B6 , oxalic acid and C | CKD and healthy controls | 29+/- 5 yrs  47 yrs +/-4 yrs  35+/- 6 yrs | 15 Healthy,  12 polyuric stage of CKD  15 CKD | Na Cl restriction in diet for 2 days and then high Nacl intake 15g/d  Intravenous furosemide challenge 20mg | Urine collection 3hr pre and post iv furosemide | In CKD significantly increased urinary excretion of B6, vitamin C and oxalic acid  High Nacl in CRF did not increase excretion of vitamins.  No increase in excretion with low prot (35g/d) and high Na Cl |  | Increase half life of furosemide in CKD from 1-2.5hrs to 6 hrs can be up to 14 hrs in literature. |
| Lee EY 1999 [[21]](https://www.zotero.org/google-docs/?ngwzpH)  Cross-sectional | Korea | B9, B12 | Dialysis | Adult | 94  34 PD  60 HD  not on supplements | Evaluation of vitamin status HD vs PD | Serum | Serum B9, B12  and red cell B9 significantly higher in PD.  PD 33 normal serum B9 levels 1 below normal - all had normal red cell B9  HD - 17 had normal serum B9 levels, 43 had below. No significant difference of serum B9 levels pre and post HD |  | ? need to look at red cell B9 for more accurate measurement of status  ate is low |

| Leblanc M  2000 [[22]](https://www.zotero.org/google-docs/?x6Nz2P)  Cross-sectional | Canada | B9 and B6 | HD | 57 +/- 13 yrs | 36 | Evaluation of vitamin status in HD patients. | Serum samples pre and post HD for P5P and B9  B12 just pre HD | Significant reduction in B9 and P5P *post HD*  26% +/- 16 % and 28%+/-14% | ? fasted samples | Pyridoxin phosphate ( converted from B6 to be active) not formed sufficiently in uremia |
| --- | --- | --- | --- | --- | --- | --- | --- | --- | --- | --- |

| Heinz J 2008 [[23]](https://www.zotero.org/google-docs/?vtzEXs)  Cross-sectional | Germany | B1,B2, B6, B9 and B12 | HD | Adult | 30  (15 high flux 15 low flux) | Evaluation of vitamin status of patients on high or low flux HD. | Serum pre and post HD | Decrease seen 37% B9, 35% B6, 6% B1, 7% B2.  No significant reduction in B12 and RBC B9  No significant difference between HF and LF similar results for both. |  |  |
| --- | --- | --- | --- | --- | --- | --- | --- | --- | --- | --- |
| Jung U 1998 [[24]](https://www.zotero.org/google-docs/?rstrXt)  Trial | Germany | B7 | HD  Tx  CRP | Adult | 23 HD, 23 CKD, 22 transplant, 40 healthy controls | Supplementation  B7. | Serum pre and post HD and 44 hours post.  Serum and 24 urine in the other groups during 2 weeks supplementation and 2 weeks after withdrawal.  Dietary intake by 7 day food intake diary. | Unsupplemented HD had a significantly lower intake of B7 serum levels were elevated. Supplemented HD serum levels were more elevated.  B7 serum levels dropped by 30% in supplemented HD pts and 33% in unsupplemented - after 44 hours 97-99% of basal level reached. Those on higher dose of supplement had significantly higher biotin level pre, post and 44 hrs after HD | Biotin supplement given 1 hour pre  HD. |  |
| Lasseur C 2001 [[25]](https://www.zotero.org/google-docs/?zoFiHh) Trial | France | B9, B12 | HD | 61+-12 yrs, | 12 | High-flux/high-efficiency dialysis vs contemporary dialysis  1.unsupplemented contemporary HD  2.unsupplemented HF/HE  3.supplemnted 5 mg HF/HE | Serum | B9 levels normal at the beginning of study, fell after switching to HF/HE dialysis but still within reference range, rose again after supplement to above reference range. B12 levels unaffected. |  |  |
| House AA 2000 [[26]](https://www.zotero.org/google-docs/?jgKtqq)  Trial | Canada | B12, folic acid | HD | Adult | 48 | High-flux or low-flux HD for 3 months. Multivitamin 1 mg B9, 10 mg B6, 6 mg B12 daily. | Measured erythrocyte B9 and B12. | No differences in B12 or B9 levels between groups, no patients with blood levels below reference range.  At the end of study, B12 in high-flux 405 +- 181 pmol/L, low flux 484 +- 226 pmol/L. Folic acid high flux 2641 +- 896 nmol/L low flux 2707 +- 1097 nmol/L. | No report on acute effects of dialysis on B12 or B9 levels, only levels after 3 months of study. |  |
| Mudge DW et al 2005 [[27]](https://www.zotero.org/google-docs/?kD4hxW)  Trial | Australia | B6, B9, B12 | HD | 58.0±14.8 yrs | 23 | Supplementation  B6 25 mg B9  acid 5mg daily | Serum | There was no significant  difference between red cell B9, vitamin B6, vitamin  B12 or albumin levels between HD membranes. |  |  |
| Kopple JD 1981 [[28]](https://www.zotero.org/google-docs/?bYg2tM)  Trial | USA | B6 | CKD, HD, PD | Adult | Controls 17,  CRF 37, PD 16, HD 55 | 1.Supplemented  2.different dosages 1 - 41 mg/day for 21-250 days | Erythrocyte glutamic pyruvic transaminase activity (EGPT stimulated/basal; value 1.25 or less was considered normal) in the morning or before + after HD | 1.High incidence of B6 deficiency. EGPT index was significantly greater than normal in each group of nondialyzed patients with CRF and in the PD and HD patients. Higher in PD and HD than CRF. B6 deficiency seen in CRF despite sufficient dietary intake.  2.Fluctuation in normal/abnormal levels over time. B6 deficiency can be corrected with 10-50 mg/day pyridoxine hydrocholride (8.2 or 41 mg pyridoxine). Lower supplementation did not correct deficiency in all patients. In PD patients, 2.5 mg/day was sufficient. 10 mg needed if the patient is catabolic or receives B6 antagonistic medication. | Did not report pre and post HD values |  |
| Cunningham JC 1981 [[29]](https://www.zotero.org/google-docs/?iSWbUY)  Cross-sectional | UK | B9 | HD | Adults | 15 | Evaluation of vitamin status in HD patients. | Serum samples from afferent and efferent dialyser lines 30 min + 6.5 hrs into dialysis | The mean afferent dialyser line ("arterial") serum B9 concentration  fell significantly from 4.31 ±0.47 to 3.49±0.38 µg/l during the six  hours' dialysis (p<001). The median loss of B9 into the dialyser was  37.3 µg (range 3.6-91.5 µg) per dialysis session (vs urinary loss of 10 µg in healthy). No iron deficiency in those who had low serum B9 levels. |  |  |
| Ramirez G et al 1986 [[30]](https://www.zotero.org/google-docs/?gzE4s9) Trial | USA | B1, B3, B6, B9,B12, vit C | HD | 58+-11 yrs | 15 | Supplementation per day:  200 mg C  15 mg B1  10.2 µg B2  50 mg B3,  5 mg B6,  10 mg B5,  1 mg folic acid; supplementation stopped at initiation of study | Serum B vitamins every 1 month for 6 months, then 2-monthly; microbiological assays  C serum pre, during and post dialysis | Vitamin B levels remained mainly within reference range despite discontinuing supplementation; low levels for some for B9, B6, B1 and B3.  Vit C : Mean SD (N = 21) pre HD 9.29 +- 4.0, during HD 8.64 +- 5.1, post HD 8.58 +-4.24; lower than in controls but within reference range |  |  |
| Frank T 2000 [[31]](https://www.zotero.org/google-docs/?0UN1mv)  Trial | Germany | B1 | CKD, HD and Transplant  Tx 19 | Adult | 57  Plus 1900 in control grp as ref value | HD – supplemented 1.5-8.0 mg a day x 3 a week post HD session – random selection of supplementation | Semi quantitative diary – 7 day – analyzed with computer software  Fasted serum samples and 24 hr urine after 7 days for HD also pre, post and 44 hr after HD serum also checked. Additional 2 day diary also kept starting after the HD session and ending before the next session. 14 after the end f vit supp serum were repeated with HD group | HD mean intake without supp 0.83mg+/- 0.3mg/d – lowest of all groups  % that did not meet RDA for B1 ,32% transplant, 43% CKD, 82% HD  Serum- HD on 8mg supp had the highest serum levels and the transplant the lowest but no significant difference  EDK marginal deficiency seen in all groups highest seen in HD on 8mg and Transplant – no significant difference.  Dialyzer ( low vs high flux) type, duration exerted influence |  |  |
| Ubukata M 2015 [[32]](https://www.zotero.org/google-docs/?xf613v)  Cross-sectional | Japan | B1 | HD | Adults | 100 | Evaluation of vitamin status on HD | Serum B1 pre and post HD | Average level 50.1 +/- 75.9(normal range 24-66 ng/mL), 15 pts showed levels < 2418 pts post HD levels lower then pre. |  | Serum levels of AST aspartate aminotransferase and ALT- alanine amino transferase were sig correlated with serum levels of thiamine and suggest should be used as a marker of low serum levels of thiamine |
| Bevier A et al 2022  Cross-sectional  cro | Australia | B1,B2,B6,B9,B12,C | HD | Adults | 39 | Evaluation of dialysate losses  and vitamin status in HD patients. | Serum pre and post dialysis | Serum values significantly decreased for B1 (20.2%), B2 (13%), B6 (25.4%),  B9 (32.6%), C (66.6%) and selenium (6.7%), B12 not affected.  Dialysate losses per session: 1.12 +- 0.88 mg for B1, 0.28 +- 0.30 mg for B2,0.33 +- 0.09 mg for B6, 0.3 +- 0.18 mg for B9, 147.5 +- 145.50 mg for C. |  |  |

1. [DoH, ‘Dietary Reference Values for Food Energy and Nutrients for the United Kingdom’; IoM, ‘Dietary Reference Intakes for Vitamin A, Vitamin K, Arsenic, Boron, Chromium, Copper, Iodine, Iron, Manganese, Molybdenum, Nickel, Silicon, Vanadium, and Zinc.’; EFSA, ‘EFSA NDA Panel (EFSA Panel on Dietetic Products, Nutrition and Allergies), 2013. Scientific Opinion on Nutrient Requirements and Dietary Intakes of Infants and Young Children in the European Union.’; NNR, ‘Nordic Nutritional Recommendations 2012’; NHMRC, ‘Nutrient Reference Values for Australia and New Zeland. Including Recommended Dietary Intakes’.](https://www.zotero.org/google-docs/?7C7lGX) [↑](#footnote-ref-1)
2. [IoM, ‘Dietary Reference Intakes for Vitamin A, Vitamin K, Arsenic, Boron, Chromium, Copper, Iodine, Iron, Manganese, Molybdenum, Nickel, Silicon, Vanadium, and Zinc.’; DoH, ‘Dietary Reference Values for Food Energy and Nutrients for the United Kingdom’; EFSA, ‘EFSA NDA Panel (EFSA Panel on Dietetic Products, Nutrition and Allergies), 2013. Scientific Opinion on Nutrient Requirements and Dietary Intakes of Infants and Young Children in the European Union.’; NNR, ‘Nordic Nutritional Recommendations 2012’; NHMRC, ‘Nutrient Reference Values for Australia and New Zeland. Including Recommended Dietary Intakes’.](https://www.zotero.org/google-docs/?XL3dj6) [↑](#footnote-ref-2)
